# Cardiovascular disease progression in female Zucker Diabetic Fatty rats occurs via unique mechanisms compared to males

**DOI:** 10.1038/s41598-017-18003-8

**Published:** 2017-12-19

**Authors:** Kelly Lum-Naihe, Ryan Toedebusch, Abuzar Mahmood, Jamal Bajwa, Terry Carmack, Senthil A. Kumar, Sivakumar Ardhanari, Vincent G. DeMarco, Craig A. Emter, Lakshmi Pulakat

**Affiliations:** 1 Department of Medicine, University of Missouri, One Hospital Drive, Columbia, MO 65212 USA; 2 Department of Biomedical Sciences, University of Missouri, 1600 E Rollins, Columbia, MO 65201 USA; 3 Department of Nutrition and Exercise Physiology, Universtiy of Missouri, 204 Gwynn Hall, Columbia, MO 65211 USA; 4 Dalton Cardiovascular Research Center, University of Missouri, 134 Research Park Drive, Columbia, MO 65201 USA; 5 Harry S. Truman Memorial Veterans’ Hospital, Columbia, MO 65201 USA

## Abstract

Population studies have shown that compared to diabetic men, diabetic women are at a higher risk of cardiovascular disease. However, the mechanisms underlying this gender disparity are unclear. Our studies in young murine models of type 2 diabetes mellitus (T2DM) and cardiovascular disease show that diabetic male rats develop increased cardiac fibrosis and suppression of intracardiac anti-fibrotic cytokines, while premenopausal diabetic female rats do not. This protection from cardiac fibrosis in female rats can be an estrogen-related effect. However, diabetic female rats develop early subclinical myocardial deformation, cardiac hypertrophy via elevated expression of pro-hypertrophic miR-208a, myocardial damage, and suppression of cardio-reparative Angiotensin II receptor 2 (*Agtr2*). Diabetic rats of both sexes exhibit a reduction in cardiac capillary density. However, diabetic female rats have reduced expression of neuropilin 1 that attenuates cardiomyopathy compared to diabetic male rats. A combination of cardiac hypertrophy and reduced capillary density likely contributed to increased myocardial structural damage in diabetic female rats. We propose expansion of existing cardiac assessments in diabetic female patients to detect myocardial deformation, cardiac hypertrophy and capillary density via non-invasive imaging, as well as suggest miR-208a, AT2R and neuropilin 1 as potential therapeutic targets and mechanistic biomarkers for cardiac disease in females.

## Introduction

Extensive clinical observations over the past decade have linked diabetes to cardiovascular disease (CVD) and concluded that an estimated 70% of the diabetic population die from CVD^1^. In the non-diabetic population, women typically develop CVD nearly a decade later than men. However, this sex difference in CVD is not seen after menopause, leading to the notion that estrogen ‘protects’ women from developing CVD. Importantly, results from the 20 year Framingham Study show that diabetic women, independent of age, are at significantly higher risk of developing CVD compared to age-matched men, which suggests a deviation from the theory that women are ‘protected’ from CVD due to elevated estrogen^2,3^. In fact, subsequent long-term clinical studies show that diabetic women are an increased risk-group for CVD compared to age-matched non-diabetic women and diabetic men^4–13^. The mechanisms underlying the increased CVD risk in diabetic women and their poorer outcomes, compared to their male counterparts, are unknown and require further characterization.

In order to investigate these mechanisms, as well as better characterize the biological sex-based differences in diabetes and CVD development, we compared several key cardiac functional and metabolic parameters between four cohorts comprised of healthy male and female Zucker Lean (ZL) rats and hyperglycemic male and female Zucker diabetic fatty (ZDF) rats. ZL males (ZL-M) and ZDF males (ZDF-M) have been extensively characterized^14–17^. While ZDF-M have similar body weights and heart weights compared to ZL-M, ZDF-M exhibit a reduction in left ventricular mass and diastolic dysfunction with preserved ejection fraction^14–17^. Unlike ZDF-M that develop hyperglycemia on normal chow, ZDF females (ZDF-F) do not develop hyperglycemia on normal chow, despite being hyperphagic and obese. In this context, ZDF-F are similar to obese, T2DM women, who are also more resistant to hyperglycemia than their age-matched male counterparts^18^. To develop hyperglycemia, ZDF-F require an additional intervention, and need to be fed a high-fat chow (diet#12468) diet^19^. However, to date there are no studies that provide a detailed characterization of cardiac parameters of hyperglycemic ZDF-F, or a comparison of cardiac disease in hyperglycemic ZDF-M and ZDF-F.

Clinical studies indicate that obese diabetic women are at increased risk for congestive heart failure or death after heart attack^20^. Cardiac histopathology shows that there are significant increases in myocardial hypertrophy in obese women with no symptoms of cardiac disease^10^. Therefore, we hypothesized that ZDF-F suffers from increased myocardial structural damage that would not be identified by standard clinical diagnostic procedures. We also expected that differences in cardiac structural damage between ZDF-F and ZDF-M render ZDF-F more vulnerable to CVD. Therefore, we examined whether there are any differences between cardiac structural parameters, such as the extent of hypertrophy, fibrosis, and myocyte loss between young male and female diabetic rats. Mechanistically, we anticipated a biological sex-based difference in several biomarkers our group and others have previously identified as causal for the differences in the progression of diabetes-induced CVD. Table 1 outlines a list of these biomarkers along with our rationale.

**Table 1:** Rationale for the biomarkers used in this study.

Biomarker	Rationale for Investigating Cardiac Expression Pattern
*Intracardiac cytokines*	Suppression of intracardiac anti-fibrotic cytokines in diabetic male Zucker Obese rats is implicated in cardiac fibrosis^25^
*Agtr2* (AT2R)	Anti-inflammatory and promotes cardiac repair. Sex-bias in expression profile^30–35^.
*Med13* (MED13)	Prevents weight gain, improves insulin sensitivity and cardiac function^36,37^.
miR-208a	Suppresses *Med13*, and promotes cardiac hypertrophy^38,39^.
miR-29a, b, & c	Blood-based biomarker for T1DM and T2DM and promotes cardiac disarray^40–42,64–66^

**Figure 1:**
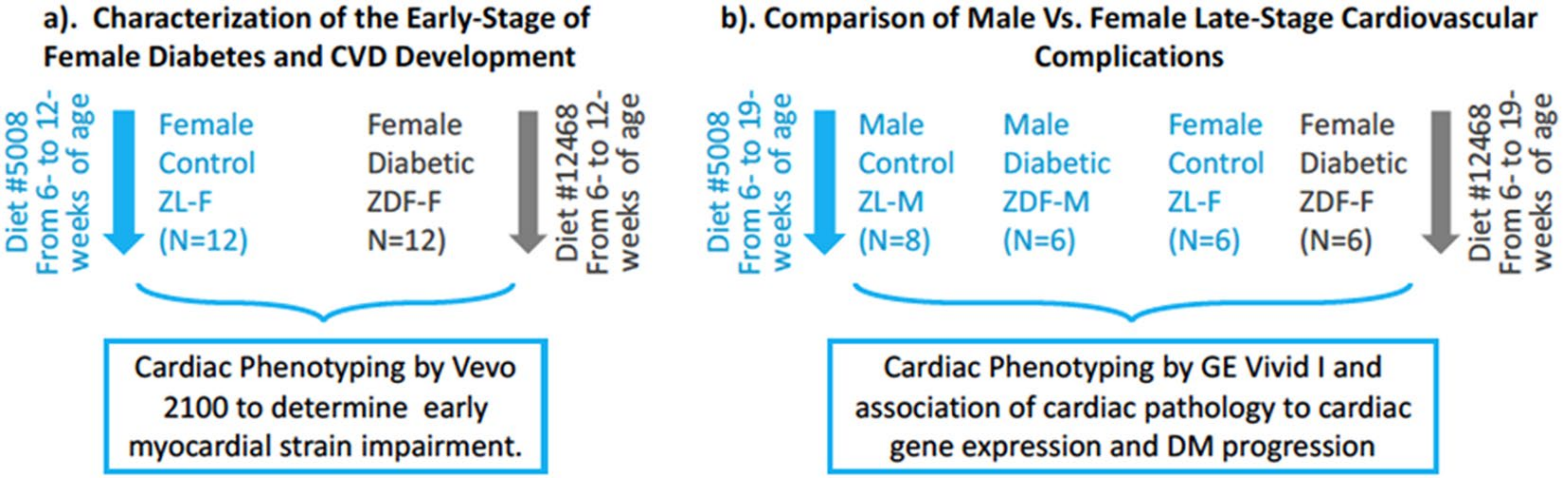
Characterization of disease progression and identification of sex differences in late stage diabetic cardiomyopathy. Depicted in this figure are the two experiments carried out to understand early stage and sex specific cardiovascular disease in diabetic rats. (**a**) Early stage female diabetic rats were studied to understand cardiac strain impairment. (**b**) Outline of the late stage male versus female cardiovascular complications secondary to diabetes.

We reported recently that there are significant differences in cardiac cytokine proteins between healthy and diabetic male rats^21^. To our knowledge, there are no reports that define biological sex differences between cardiac cytokines of healthy and diabetic rats. Here we compared differences between 67 cardiac cytokine proteins of healthy and diabetic male and female rats. This study is the first to compare cardiac function, histopathology, intracardiac cytokine profile and metabolic analysis between ZL-F and ZDF-F, and between ZDF-F and ZDF-M at the ages of 3- and 5-months as outlined in Fig. 1.

## Results

### Metabolic phenotype of ZDF-F and ZDF-M at the age of 3- and 5-months

**Figure 2:**
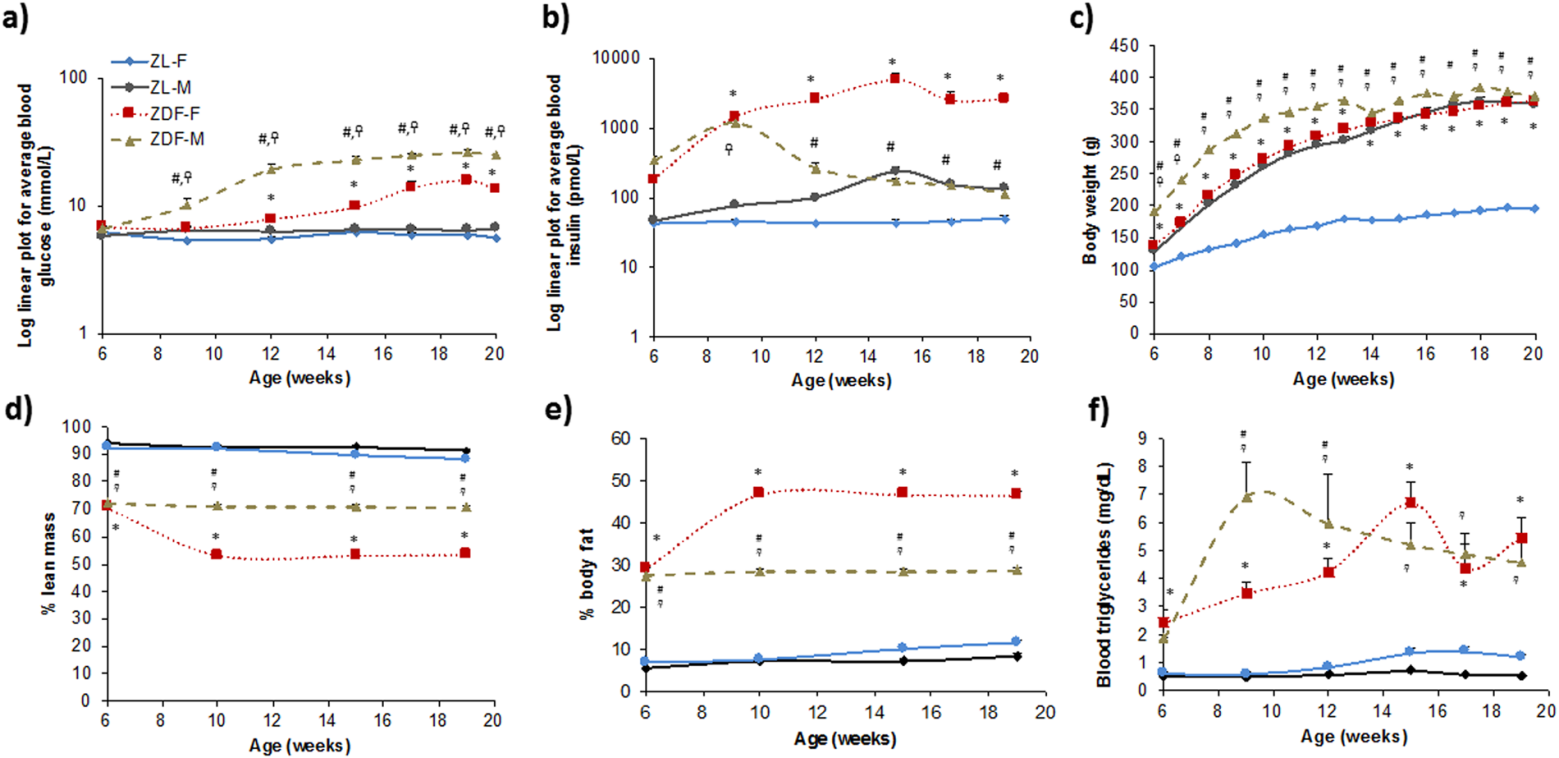
Changes in fasting blood glucose, serum insulin, and triglyceride levels, body weight, percentage of body fat, and lean muscle mass of healthy and diabetic male and female rats. Six-hour fasting blood collection at the indicated ages was performed for analysis of (**a**) plasma glucose, (**b**) serum insulin, and (**f**) serum triglyceride levels using commercially available assays (Beckman-Coulter, Brea, CA) on an automated clinical chemistry instrument (AU680, Beckman-Coulter, Brea, CA). Insulin was measured by an ELISA kit specific for rat insulin. (**c**) Body weight was determined weekly and monthly. (**d** and **e**) Body composition measurements were performed using the EchoMRI 4in1/1100. Values are means ± SEM. n = 6 for ZL-F, ZDF-F, and ZDF-M, and n = 8 for ZL-M. *p < 0.05 vs. ZL-F, ^†^p < 0.05 vs. ZL-M, and ^#^p < 0.05 vs. ZDF-F using two-way ANOVA.

ZL-M and ZL-F did not show significant changes in fasting plasma glucose, insulin or triglyceride levels at the ages of 12-weeks (3-months) or 19-weeks (5-months) (Fig. 2a,b and f). ZDF-M exhibited hyperglycemia that worsened with age (Fig. 2a). ZDF-M were also hyperinsulinemic initially, but insulin levels decreased by the age of 12-weeks (Fig. 2b). These observations are consistent with previous reports^14,16^. ZDF-F became hyperglycemic at 12-weeks of age and hyperglycemia worsened despite hyperinsulinemia as they got older (Fig. 2a). ZDF-F were severely hyperinsulinemic throughout the study, although insulin levels slightly dropped at the age of 17-weeks (Fig. 2b). ZDF-F had significantly higher body weight than ZL-F, but the lowest percentage of lean muscle mass (Fig. 2d) and the highest percentage of body fat (Fig. 2e) among the four groups. Thus, ZDF-F on diet#12468 developed hyperglycemia with severe hyperinsulinemia at the age of 3-months and exhibited obesity and metabolic syndrome.

### Cardiac phenotype of ZDF-F and ZDF-M at 3- and 5-months of age

Supplemental Table S1 shows comparison of cardiac functions of all four groups at 3- and 5-months of age using a GE Vivid I Ultrasound system. Fractional shortening was greater than 50% at both time points, and heart rate/stroke volume were largely similar, providing evidence of compensated systolic function in all groups. ZDF-F and ZDF-M exhibited an increase in isovolumic relaxation time, indicative of impaired early diastolic relaxation, and this effect was greatest in ZDF-M. The ratio of early to late blood flow through the mitral valve (E/A ratio), measured using pulse wave Doppler, was significantly reduced in ZDF-F. A decrease in the E/A ratio represents a shift in the early and late contributions to diastolic filling^22^. This change was clearly evident in ZDF-F (simple effect, ‡p < 0.05 vs. ZL-F at same time point), but harder to interpret in ZDF-M given males demonstrated a lower E/A ratio in general irrespective of disease. These observations are consistent with the idea that diabetic rats in both sexes have diastolic dysfunction at the age of 5-months.

Previous studies have shown that ZDF-M do not exhibit cardiac hypertrophy at any age (11-week, 16-week or 36-week), and also that LV mass was lower in ZDF-M compared to ZL-M^14,23^. However, ZDF-F differed from ZDF-M in this regard. At the age of 3-months, ZDF-F exhibited an increase in heart weight adjusted to tibia length (ZL-F:0.217 ± 0.007 g/mm; ZDF-F0.246 ± 0.009 g/mm; p < 0.05; n = 5 per group). Therefore, to gain a better understanding of the early cardiac deformation and dysfunction in ZDF-F, we analyzed a second cohort of ZL-F and ZDF-F using the more sensitive Vevo® 2100 platform.

### Cardiac phenotype of 3-month-old ZDF-F and ZL-F shows early diastolic and systolic dysfunction in ZDF-F

**Figure 3:**
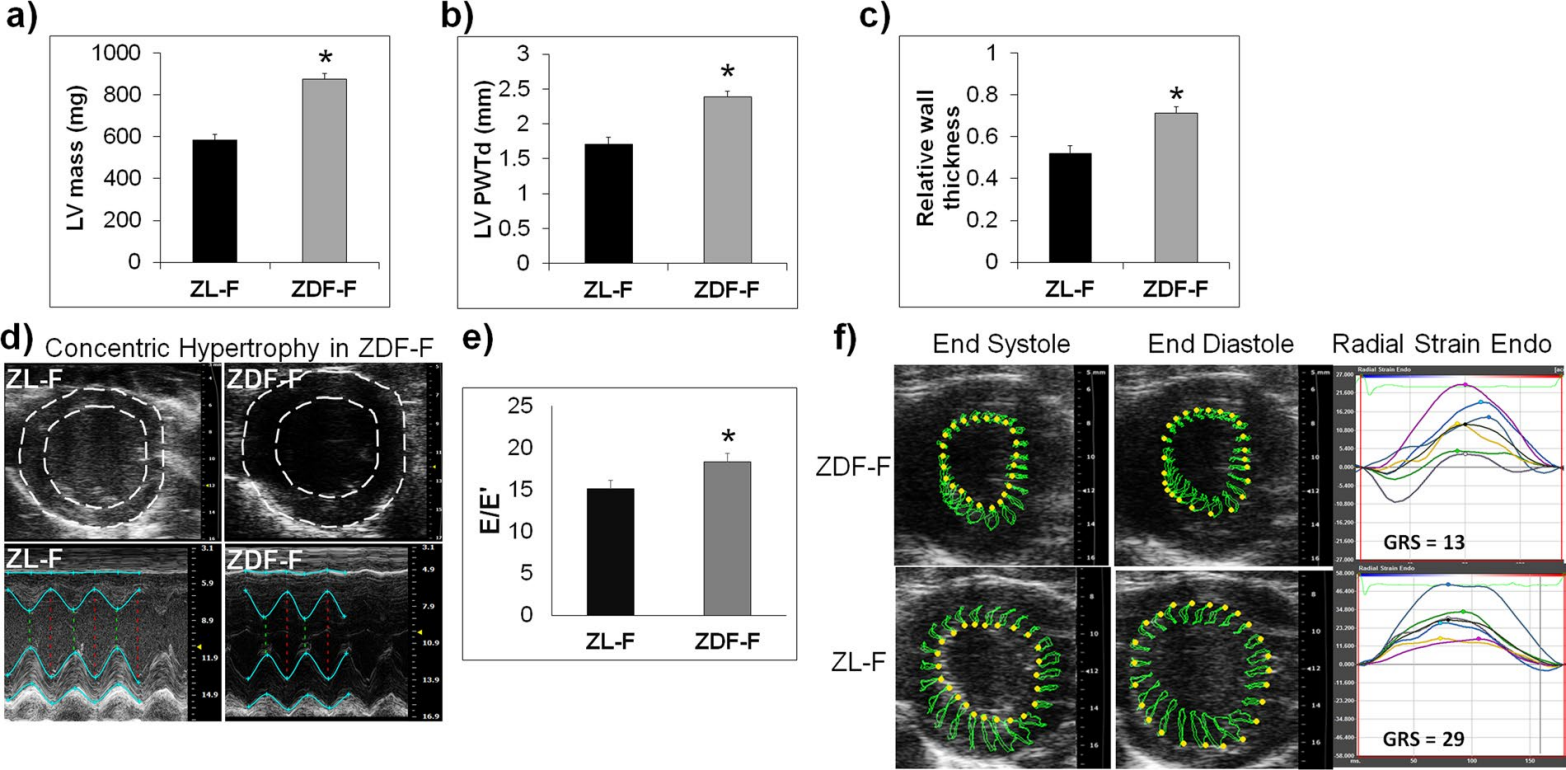
Cardiac function and myocardial strain analysis in 3-month old ZL-F and ZDF-F rats. Graphs show comparison of (**a**) left ventricular (LV) mass, (**b**) LV posterior wall thickness during diastole (PWTd), and (**c**) LV relative wall thickness (RWT) calculated by using the formula (PWTd + AWTd)/LVIDd - where AWT is the anterior LV diastolic wall thickness and LVID is the LV internal diameter at end diastole. (**d**) Concentric hypertrophy in 3-month old ZDF rats. Top ultrasound panels: B-mode images in short axis view at the level of the papillary muscles and at end diastole. Bottom ultrasound panels: Representative M-mode spectra showing thicker LV anterior and posterior walls in ZDF hearts. (**e**) Graph shows E/E’, a measure of diastolic LV filling pressure and a powerful predictor of primary cardiac event in humans^25^. (**f**) Speckle tracking analysis of strain in ZDF-F and ZL-F rats. Top panels are representative images from a ZDF rat taken at end systole and indicate an abnormal pattern of deformation of the endocardium during the cardiac cycle compared to those from a ZL rat (lower panels). Regional strain curves for each rat are displayed to the right and values for global radial strain (GRS) are shown in the panels. For echocardiography data in (**a–c)** and (**g)**, n = 10–12 per group; for (**d–f)**, n = 5 per group. Values are means ± SEM. *p < 0.05 for ZDF-F vs. ZL-F as determined by unpaired *t*-test.

Analysis of a second cohort of ZL-F and ZDF-F rats using the Vevo^®^ 2100 platform equipped with a 30Mz high-frequency transducer showed that 3-month old ZDF-F had an increase in left ventricular (LV) mass compared to ZL-F (Fig. 3A, p < 0.05). Moreover, compared to ZL-F, ZDF-F exhibited increased left ventricular posterior wall thickness during diastole (LVPWTd) and relative wall thickness (RWT) (Fig. 3B and C p < 0.05). Phenotypically, ZDF-F exhibited a combination of an increase in LV mass with no group differences in LV internal diastolic diameter resulting in increased RWT, an indicator of concentric LV hypertrophy (in contrast to eccentric remodeling where the LV wall thins in parallel with an increase in LV internal diastolic diameter) (Fig. 3D). E’ velocity was measured by tissue Doppler to calculate E/E’ ratio, a clinical parameter that is correlated to elevated LV end diastolic pressure in humans^24,25^. The E/E’ ratio was significantly increased in ZDF-F vs ZL-F consistent with impairment in diastolic function (Fig. 3E, p < 0.05). Speckle tracking strain analysis is a powerful tool to detect early deformation of the heart with far greater sensitivity and specificity than conventional echocardiographic measures, such as fractional shortening (FS)^26–28^. ZDF-F demonstrated significant impairments in peak endocardial radial strain and strain rate compared to ZL-F (Fig. 3F and Supplemental Table S2). Longitudinal strain was also reduced in the epicardial PLAX (parasternal long axis) view. Collectively, these data indicate the presence of diastolic dysfunction, impaired systolic mechanics, and normal fractional shortening combined with cardiac hypertrophy in 3-month old ZDF-F. These observations also highlight the utility of speckle tracking strain analysis in uncovering early myocardial deformation and systolic impairment. Taken together, ZDF-F differed from ZDF-M in that they demonstrate impaired systolic mechanics and concentric LV hypertrophy, despite having normal fractional shortening and preserved ejection fraction as ZDF-M.

### Differences in the intracardiac cytokine profiles between male and female rats

Our previous studies on ZL-M and male Zucker obese (ZO-M) rats identified that T2DM induces cardiac fibrosis and suppresses anti-fibrotic intracardiac cytokines^21^. Interestingly, we also found that Rapamycin, an immunosuppressant, promotes cardiac fibrosis and suppresses intracardiac anti-fibrotic cytokines in ZL-M^21^. To gain a better understanding of the biological sex differences in intracardiac cytokine profiles of healthy and diabetic rats, we analyzed 67 cytokines in the heart lysates of all four groups.

#### ZL-M versus ZL-F

**Figure 4:**
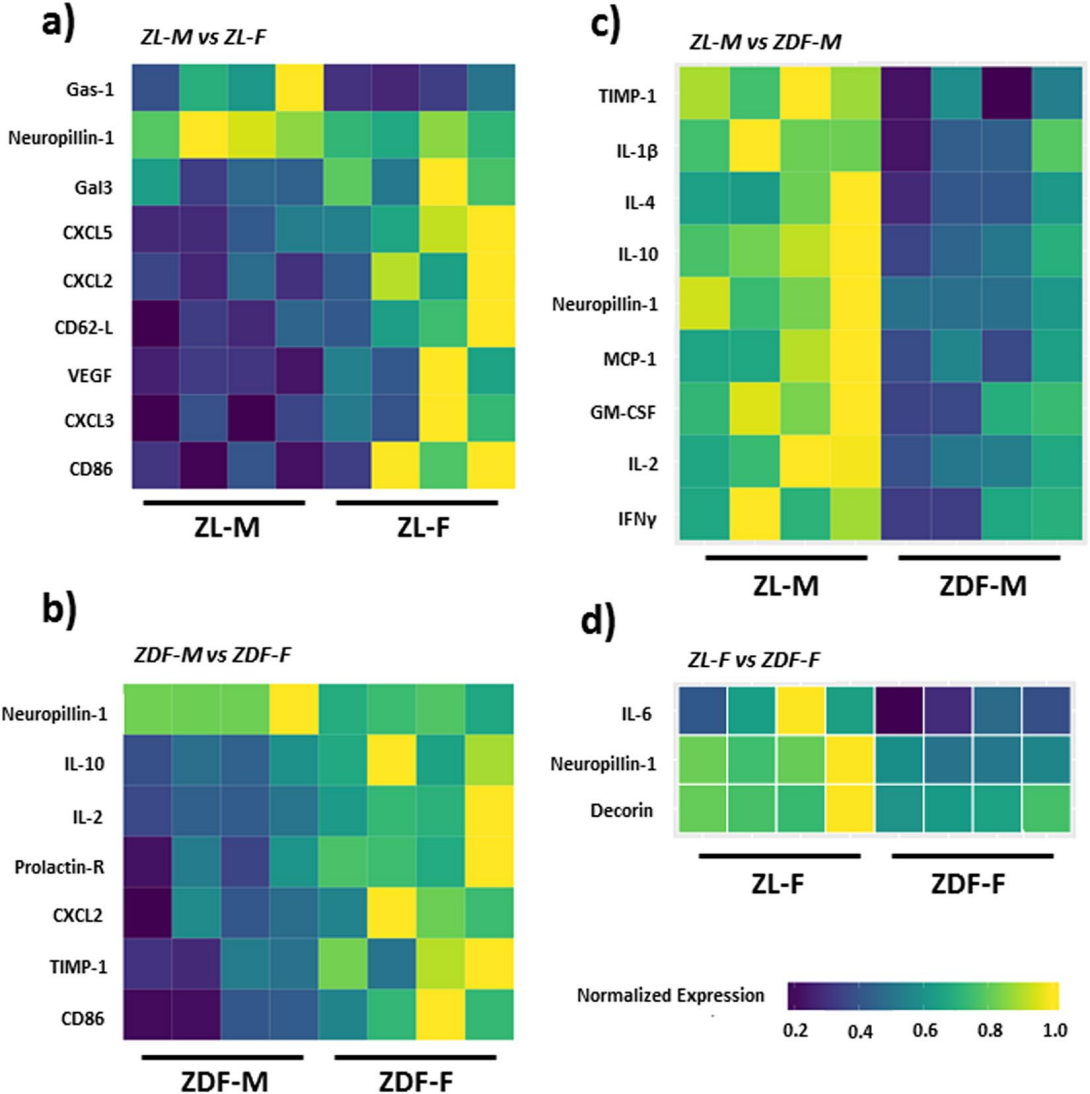
Heat maps showing differentially expressed cytokines between all groups. Each of the four comparisons are displayed as heat maps illustrating each animal for each different cytokine. (**a**) ZL-M vs. ZL-F, (**b**) ZDF-M vs. ZDF-F, (**c**) ZL-M vs. ZDF-M, (**d**) ZL-F vs. ZDF-F. Each heat map is a graphic representation of relative expression of cardiac cytokine levels with individual cardiac samples arranged along the x-axis and cytokiness along the y-axis. Yellow indicates the highest expression for each cytokine (1.0), while dark blue indicates lower expression relative to the sample with the highest expression. Expression was normalized for each cytokine across all animals (across each row). Statistical significance was determined using Student’s t-test. p < 0.05 for all proteins, n = 5 for each group.

Comparison of intracardiac cytokine protein expression between ZL-M and ZL-F showed that males had lower levels of many important cytokines (Fig. 4a and Supplemental Table S3). Out of the 67 cytokines analyzed, the following nine were significantly decreased in ZL-M compared to ZL-F (p < 0.05): CD86 (cluster of differentiation 86), CXCL2 (C-X-C motif chemokine ligand 1), CXCL3 (C-X-C motif chemokine ligand 3), CXCL5 (C-X-C chemokine ligand 5), Gal-3 (galectin 3), CD62-L (selectin L), and VEGF (vascular endothelial growth factor). Conversely, only Gas-1 (growth arrest specific 1) and Neuropilin1 were increased (p < 0.05) in ZL-M compared to ZL-F (Fig. 4a and Supplemental Table S3).

#### ZDF-M versus ZDF-F

Comparison of intracardiac cytokine expression between ZDF-M and ZDF-F showed reduced expression of several cytokines in ZDF-M compared to ZDF-F. Out of 67 cytokines, the following were significantly decreased (p < 0.05) in ZDF-M compared to ZDF-F: CD86 (cluster of differentiation 86), CXCL1 (C-X-C motif chemokine ligand 1), interleukin 10 (IL-10), interleukin 2 (IL-2), Prolactin-R, TIMP-1 (metalloproteinase inhibitor 1). Only Neuropilin1 was increased in ZDF-M compared to ZDF-F (Fig. 4b and Supplemental Table S3, p < 0.05).

### Differences in the intracardiac cytokine profiles between healthy and diabetic rats

In an attempt to better understand the effect of diabetes within sex, we compared intracardiac cytokine profiles of ZDF-M to ZL-M and ZDF-F to ZL-F.

#### ZL-M versus ZDF-M

Expression of the following 9 intracardiac cytokines were suppressed in ZDF-M (p < 0.05) compared to ZL-M: GM-CSF (colony stimulating factor 2), IFN-γ (interferon gamma), IL-10 (interleukin 10), interleukin 1 beta (IL-1β), IL-2 (interleukin 2), IL-4 (interleukin 4), MCP-1 (C-C motif chemokine ligand 2), Neuropilin 1, and TIMP1 (metalloproteinase inhibitor 1) (Fig. 4c and Supplemental Table S3). Reduction in the expression levels of anti-fibrotic GM-CSF, IFN-γ, and IL-10 in the hearts of ZDF-M compared to ZL-M is consistent with what we reported previously in male ZO rats^21^.

#### ZL-F versus ZDF-F

Decorin, IL-6 (interleukin 6), and Neuropilin1 were the only intracardiac cytokines that were significantly decreased (p < 0.05) in ZDF-F compared to ZL-F. IL-6 is implicated in chronic inflammation, fibrosis and aging^29^. Suppression of intracardiac IL-6 expression in ZDF-F compared to ZL-F implied that ZDF-F could be protected from cardiac fibrosis. Moreover, expression levels of the anti-fibrotic cytokines (GM-CSF, IFN-γ, and IL-10) were similar in both ZDF-F and ZL-F, further suggesting protection from cardiac fibrosis in ZDF-F (Fig. 4d and Supplemental Table S3).

### Bioinformatic analysis of intracardiac cytokine array predicts fibrosis and immune cell dysregulation in ZDF-M, but not in ZDF-F

**Figure 5:**
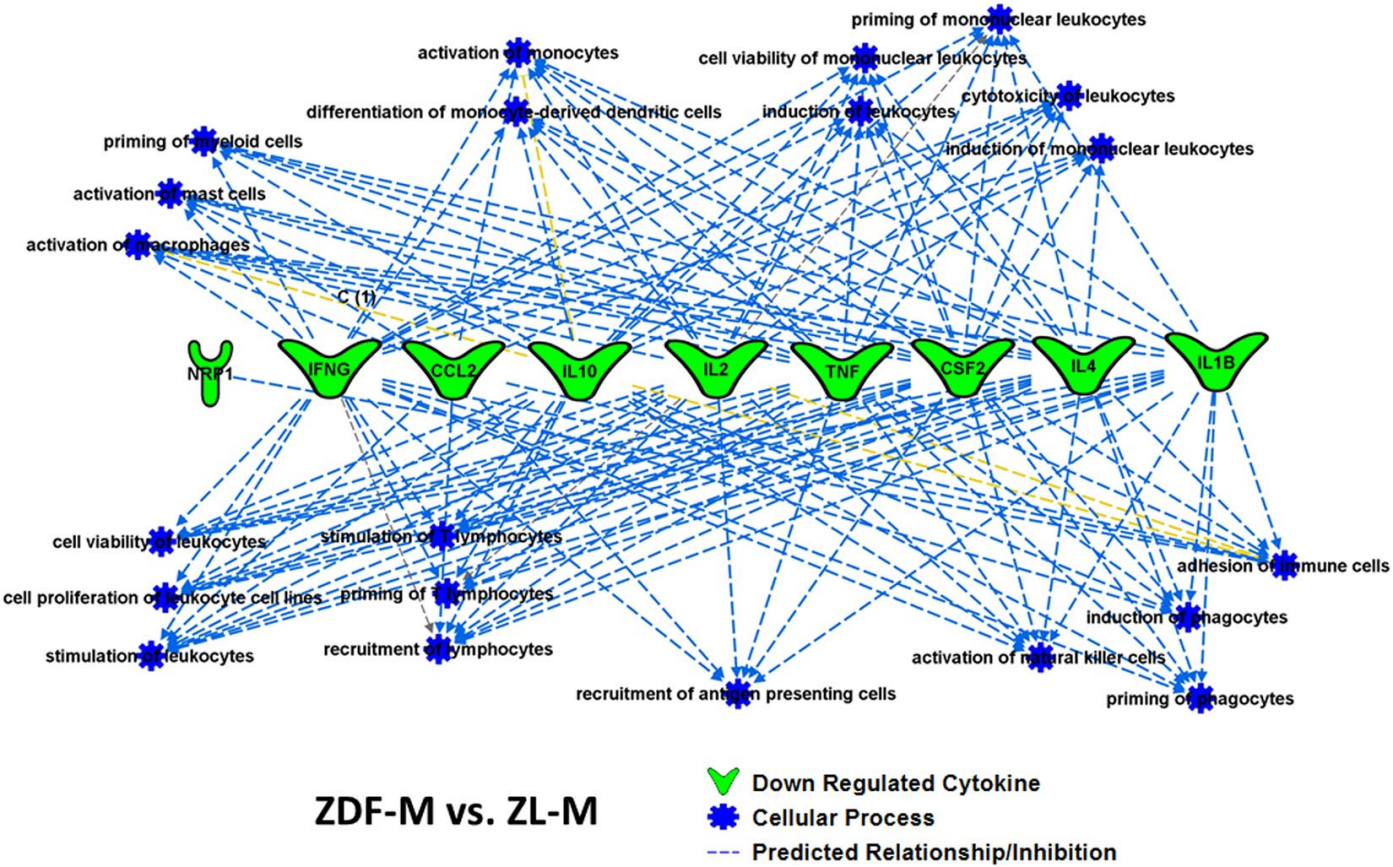
Top scoring IPA-predicted network resulting from the comparison of ZDF-M and ZL-M cytokine profiles. IPA output for cytokines that were differentially expressed between ZDF-M and ZL-M. Multiple immune cell specific functions are predicted to be inhibited based on the expression patterns of the down-regulated cytokines in ZDF-M. Green cytokines in the center indicate downregulation in ZDF-M compared with ZL-M rats. The blue connecting lines show a known relationship between the cytokine and the cellular process/function. This figure was generated using IPA’s built in feature for “Diseases and Functions”, and then selecting “immune system related” processes.

**Figure 6:**
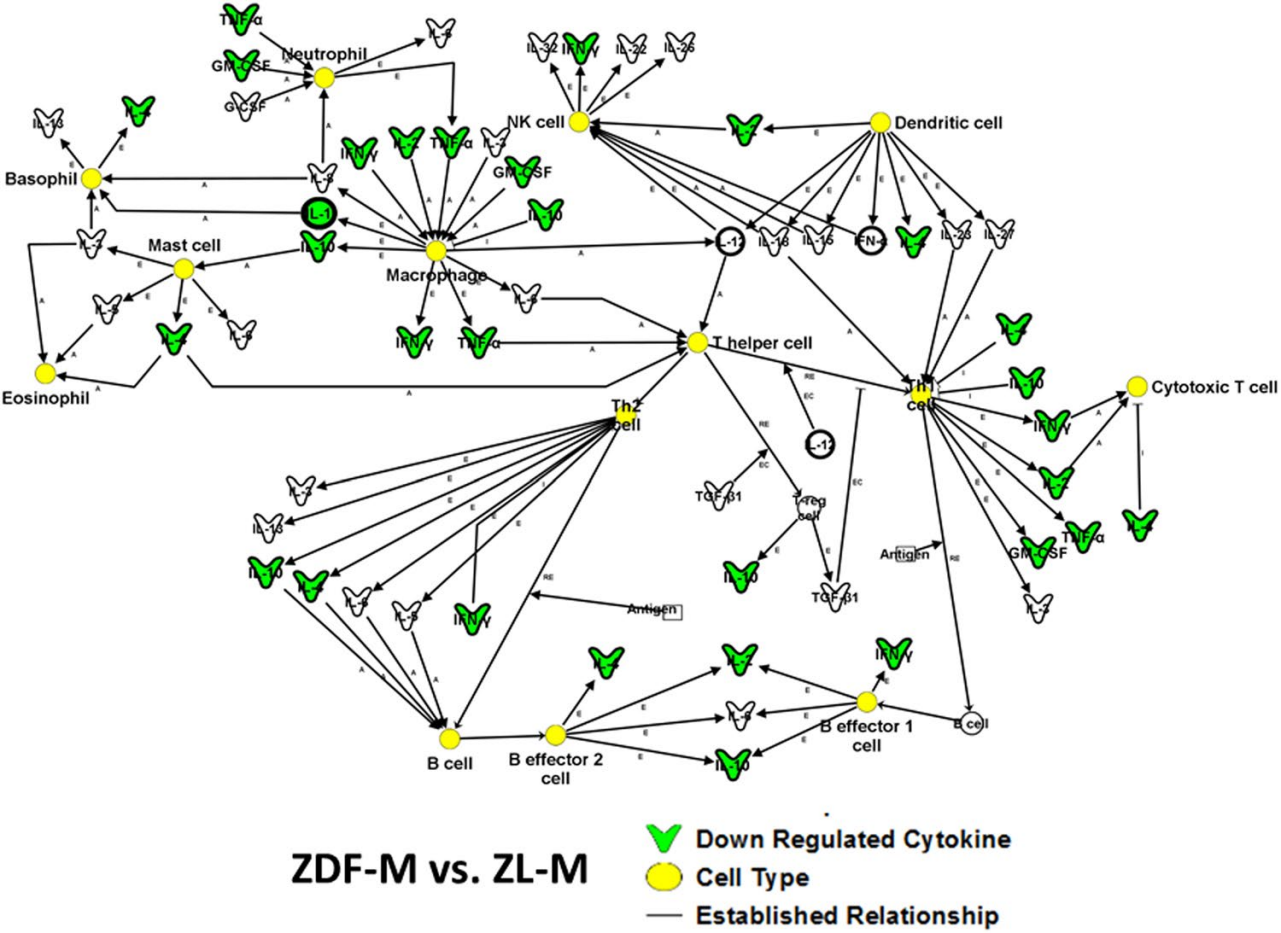
Top scoring IPA-predicted canonical pathway resulting from the comparison of ZDF-M and ZL-M cytokine profiles. The top scoring canonical pathway representing decreased cytokine expression in ZDF-M compared with ZL-M (p = 1.56E-16). Green symbols denote downregulated cytokines. Yellow circles represent the various cell types involved in the inflammatory response. White symbols are cytokines that show similar function but were not changed in our data set.

**Figure 7:**
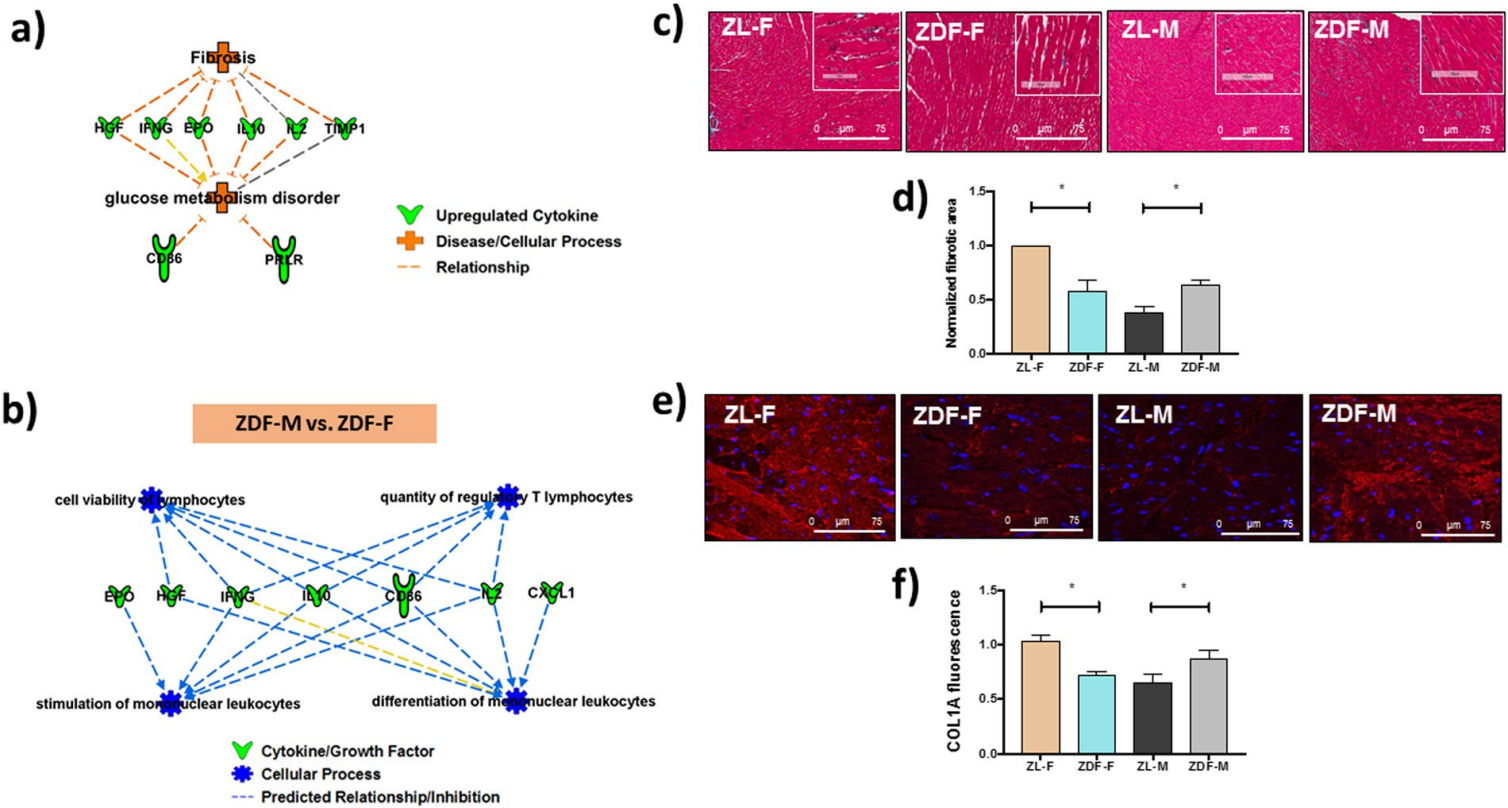
IPA-predicted activation of cardiac fibrosis and glucose metabolism disorder networks, immune dysfunction, and visualization of cardiac fibrosis in ZDF-M. IPA predicted networks show that (a) cardiac fibrosis and glucose metabolism disorder are activated, while (**b**) “cell viability of lymphocytes”, “quantity of regulatory T lymphocytes”, “stimulation of mononuclear leukocytes”, and “differentiation of mononuclear leukocytes” are inhibited. In support of cardiac fibrosis, representative images of (**c**) Trichrome stained heart sections of ZL-F, ZDF-F, ZL-M, and ZDF-M are shown. Images were taken at 10x magnification (scale bars = 75 µm). (**d**) Graph shows the normalized fibrotic area from 10 images for each animal (n = 6 animals per group). (**e**) Immunohistological staining for COL1A in heart sections of ZL-F, ZDF-F, ZL-M, and ZDF-M are shown. Images were taken at 63X magnification (scale bars = 75 µm). (**f**) Graph shows the fluorescence intensity quantified from images (n = 3–6 animals per group).

We utilized IPA (Ingenuity Pathway Analysis) software to generate relationships, pathways, and networks. This was used to elucidate how differentially expressed intracardiac cytokines between the cohorts relate to disease. The differentially expressed cytokines (p < 0.05; Gas-1, GM-CSF, IFN-γ, IL-10, IL-1β, IL-2, IL-4, MCP-1, Neuropilin 1, TIMP1) between ZL-M versus ZDF-M were used as input for IPA analysis. IPA predicted that multiple inflammatory pathways and functions were inhibited in the ZDF-M compared to ZL-M (Fig. 5). Figure 6 illustrates how different immune cells are interconnected and affected due to the expression patterns of the downregulated cytokines presented in the network. Next, 7 differentially expressed cytokines (p < 0.05; CD86, CXCL1, IL-10, IL-2, Prolactin-R, TIMP-1, Neuropilin1) between ZDF-M and ZDF-F were used as input for IPA analysis. IPA predicted that “fibrosis” and “glucose metabolism disorder” would be activated in ZDF-M (Fig. 7a). Furthermore, the following cellular-processes; “cell viability of lymphocytes”, “quantity of regulatory T lymphocytes”, “stimulation of mononuclear leukocytes”, and “differentiation of mononuclear leukocytes” were all predicted to be inhibited in ZDF-M based on IPA’s built in algorithm and statistics (Fig. 7b).

To validate the IPA prediction of increased fibrosis in ZDF-M, we analyzed the extent of cardiac fibrosis in all four groups. Analysis of the Trichrome stained heart sections via computer algorithm confirmed that interstitial cardiac fibrosis was higher in ZDF-M vs ZL-M with ZL-F and ZDF-M having more cardiac fibrosis compared to other groups (Fig. 7c and d). Immunohistochemistry analysis of Collagen 1 A antibody-stained heart sections further confirmed this observation (Fig. 7e and f). Collectively, these data suggest that cardiac fibrosis was higher in ZDF-M compared to ZL-M, but not in ZDF-F compared to ZL-F.

### Hypertrophy and myocardial disorganization between ZDF-F and ZDF-M

**Figure 8:**
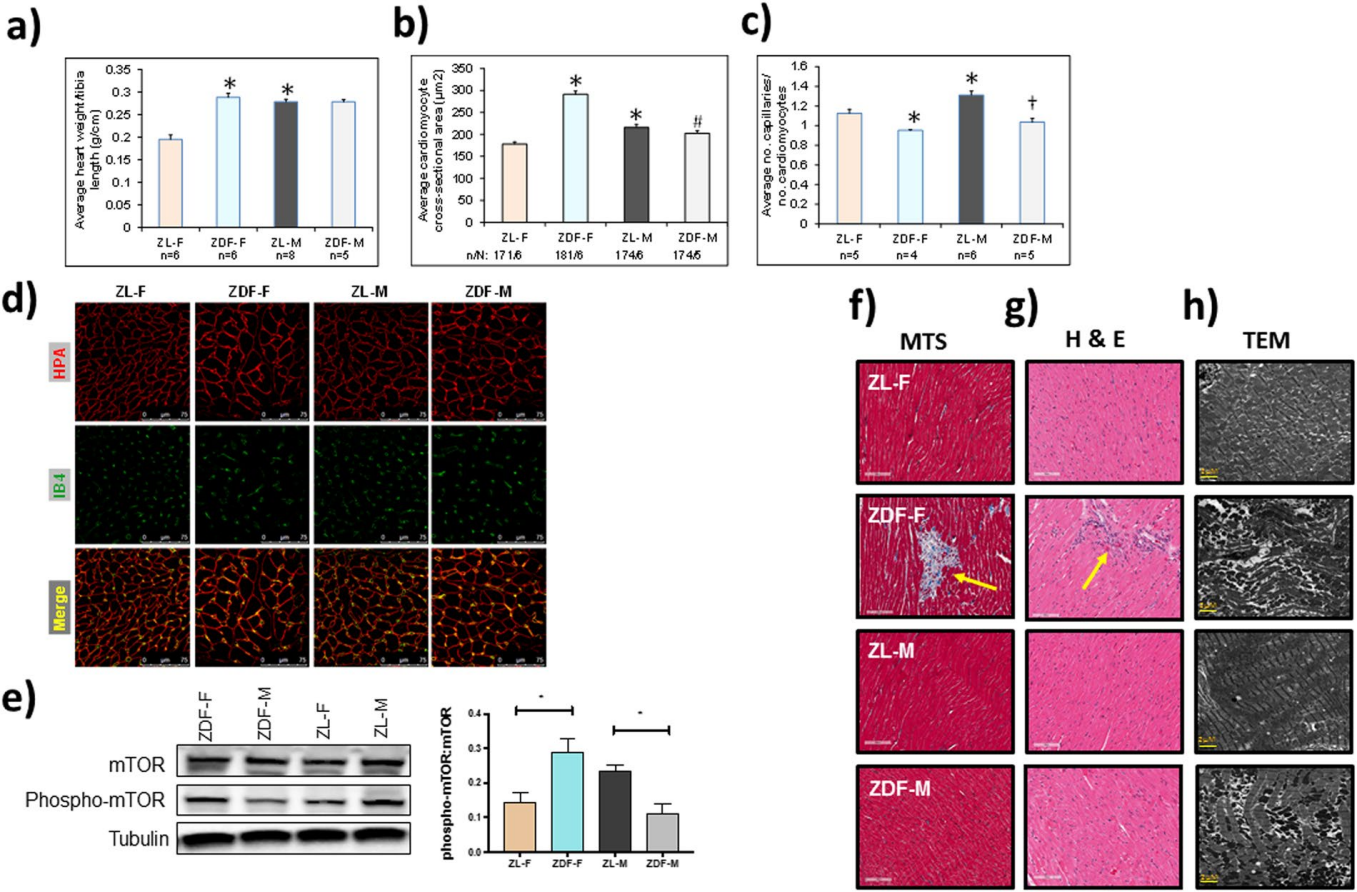
Differences between capillary rarefaction, hypertrophy and overall cardiac disorganization in ZDF-F compared to ZDF-M rats. (**a**) Graph shows heart weight adjusted to tibia length indicating ZDF-F heart is hypertrophied compared to ZL-F. (**b**) Quantification of cardiomyocyte cross-sectional area determined from HPA-staining (**c**) Quantification of capillary density determined from IB4-staining. (**d**) Representative images of heart sections stained with HPA conjugated to Alexa Fluor 647 to visualize cardiomyocyte membrane and IB4 conjugated to Alexa Fluor 594 to visualize capillaries (scale bars = 75 µm); n = 5–8 animals per group. For cardiomyocyte hypertrophy calculations, number of cardiomyocytes analyzed ranged from 171 to 181 per animal. Values are means ± SEM. *p*-values are noted and were determined by two-way ANOVA. *p < 0.05 vs. ZL-F, ^†^p < 0.05 vs. ZL-M. ZDF-F cardiomyocytes were significantly larger than any of the other 3 groups (p < 0.05). (**e**) Representative immunoblot that shows the extent of Ser^2448^phosphorylation levels of mTOR protein. Graph shows quantification of Ser^2448^phosphorylation of mTOR. n = 6 animals per group. (**f**) Representative images of cardiac tissues stained with Masson’s trichrome (MTS). Only ZDF-F exhibited scar tissue (marked with yellow arrow). (**g**) Representative images of cardiac tissues stained and hematoxylin and eosin (H&E). Yellow arrows mark blood cells spilling into interstitial regions in ZDF-F. Scale bars = 100 µm. n = 5–8 animals per group. (**h**) Representative transmission electron micrographs at 1000X showing extensive disruption of the normal cardiac organization in ZDF-M and ZDF-F compared with respective leans. n = 3–5 animals per group. Yellow scale bars = 2 µm.

At 5-months, ZDF-F continued to exhibit cardiac hypertrophy as evidenced by the significant increase in heart weight (normalized to tibia length) compared to ZL-F (Fig. 8a). Conversely, 5-month old ZDF-M did not exhibit hypertrophy and this is consistent with previous reports (Fig. 8a)^14,16^. Next, we performed histopathology analysis of formalin-fixed and paraffin-embedded 5 µm sections of heart tissues by staining with *Helix pomatia* agglutinin (HPA)^30^ conjugated to Alexa Fluor 647 to evaluate cardiomyocyte hypertrophy. We also used vascular staining with isolectin IB4^31^ to determine the capillary density in the heart. ZDF-F exhibited the most significant cardiomyocyte hypertrophy among the four groups (Fig. 8b and d). Additionally, both ZDF-F and ZDF-M exhibited significant reductions in capillary density, determined as the ratio of capillaries to cardiomyocytes, relative to lean rats. (Fig. 8c and d). Increased phosphorylation of the Ser^2448^ residue of mTOR (mechanistic target for rapamycin) that results in activation of mTOR is a hallmark of cardiac hypertrophy. As shown in Fig. 8e, we observed increased cardiac mTOR phosphorylation in ZDF-F compared to ZL-F, but not in ZDF-M compared to ZL-M. This is consistent with our previous report that shows 22-week-old ZDF-M do not have an increase in Ser^2448^ phosphorylation of mTOR^17^.

Staining of heart tissue sections with Masson’s trichrome stain (MTS) (Fig. 8f) and haematoxylin and eosin (H&E) (Fig. 8g) showed that ZDF-F had focal lesions characterized by cardiomyocyte loss and scarring that were not seen in any other groups. This damage included structural damage of the tissue as seen in MTS stained ZDF-F heart section (Fig. 8f). While four out of six ZDF-F showed similar myocardial damage, none of the ZDF-M hearts showed similar scar tissue. ZDF-F hearts also exhibited increased leukocyte infiltration as seen in the H&E stained ZDF-F heart section (Fig. 8g). This level of damage was not seen in ZDF-M hearts. ZDF-F hearts had more visible structural damage at 5-months compared to age-matched ZDF-M hearts Ultrastructural analysis with transmission electron microscopy showed that both ZDF-M and ZDF-F rats had structural disorganization of the myocardium, mitochondrial clustering, and disrupted spatial orientation of mitochondria in relation to the sarcomere compared to their lean counterparts (Fig. 8h).

### Cardioprotective Agtr2 (AT2R) expression is attenuated in ZDF-F, but not in ZDF-M, compared to their healthy age-and sex-matched counterparts

**Figure 9:**
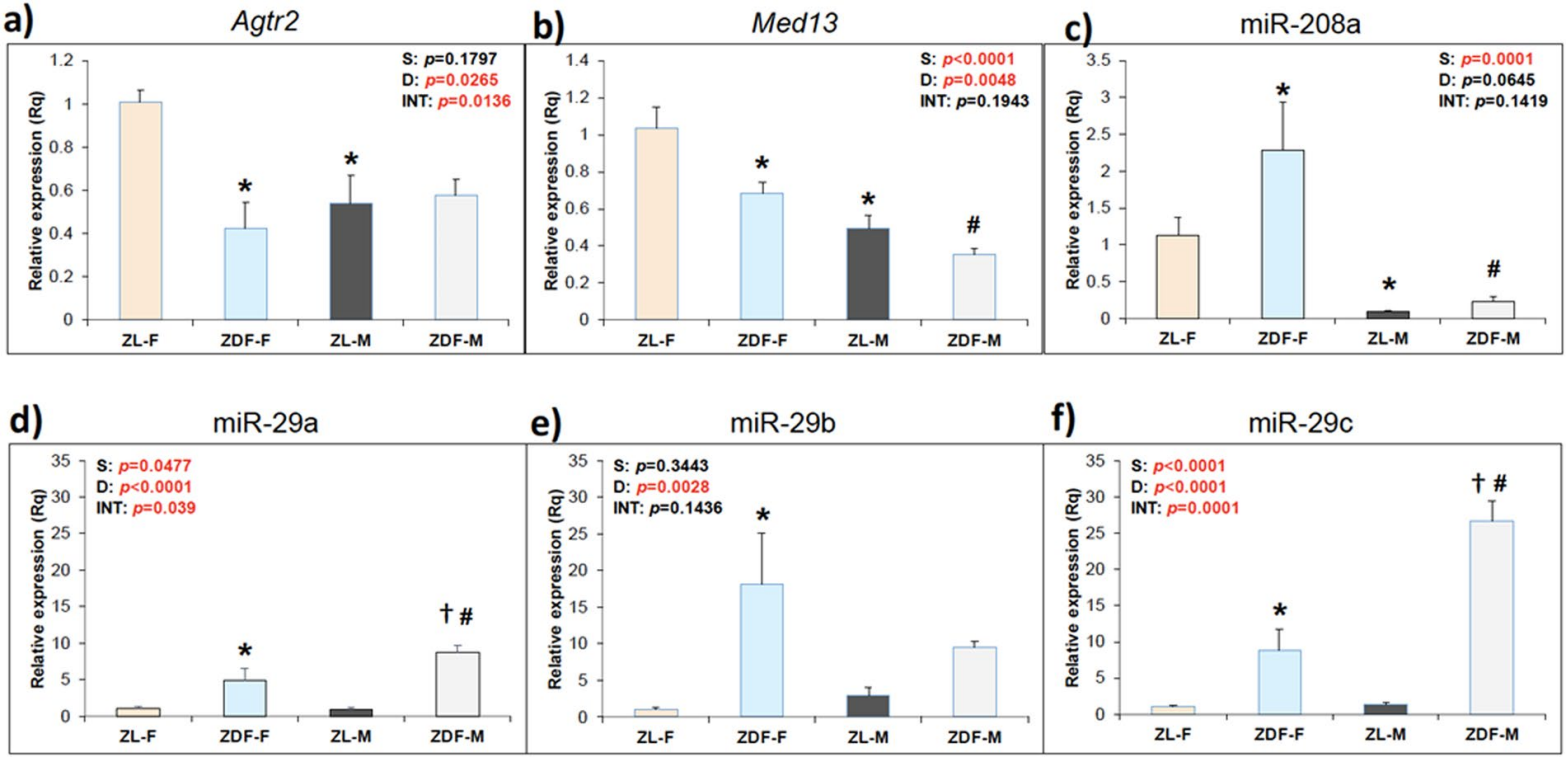
Expression patterns of cardioprotective *Agtr2* and *Med13*, and cardio-deleterious miR-208a and diabetic marker miR-29 family miRNAs in heart tissues of 5-month old healthy and diabetic, male and female rats. (**a**) Graph shows qRT-PCR data on the expression of cardiac *Agtr2* (AT2R) mRNA. ZL-F rats had a significantly higher expression of *Agtr2* mRNA compared to ZL-M rats, indicating a sex difference in the expression of cardiac AT2R. *Agtr2* mRNA expression was strongly suppressed in ZDF-F rats compared to ZL-F rats, indicating that diabetes progression leads to loss of cardioprotective AT2R expression in females. There was no significant loss of *Agtr2* mRNA expression in ZDF-M rats. (**b**) Graph shows qRT-PCR data on cardiac expression of *Med13*. ZL-F had a significantly greater expression of *Med13* mRNA than ZL-M, suggesting a sex bias. Cardiac *Med13* expression was suppressed in both ZDF-F and ZDF-M compared to lean controls, indicating a diabetes-associated suppression of *Med13*. (**c**) Graphs show qRT-PCR data on the expression of cardiac miR-208a. Cardiac miR-208a expression was elevated in both ZDF-F and ZDF-M compared to lean controls, indicating a diabetes-associated elevation of cardiac miR-208a. The miR-208a expression was higher in both ZL-F and ZDF-F compared to their male counterparts. Thus miR-208a exhibits sex bias. Interestingly, ZL-F had higher cardiac *Med13* expression than ZL-M. (**d**–**f**) Graphs show qRT-PCR data on the expression of cardiac microRNA miR-29a, b and c. Expression of all miR-29 family miRNAs were increased in ZDF-F and ZDF-M. miR-29c was the most differentially expressed miRNA between male rats (**f**) whereas miR-29b was the most differentially expressed miRNA between female rats (**e**). Additionally, miR-29b levels were higher in ZDF-F rats compared to ZDF-M rats. Thus, there were sex differences in miR-29 family miRNA expression. Number of animals per group ranged from 6–8 for ZL-M and 4–6 for all other groups. Values are means ± SEM. *p*-values are noted and were determined by two-way ANOVA*S*, sex; *D*, diabetes; *INT*, interaction. *p < 0.05 vs. ZL-F; ^†^ *p* < 0.05 vs. ZL-M; ^#^ *p* < 0.05 vs. ZDF-F.

Increased expression of *Agtr2* in murine female vasculature is implicated in protection from hypertension and CVD^32–34^. Moreover, AT2R activation has a crucial role in cardiac repair^35–37^. However, it is not known whether cardiac AT2R expression exhibits a sex bias. We found that cardiac *Agtr2* expression, as determined by qRT-PCR, was higher in ZL-F compared to ZL-M (p < 0.05). However, there was an almost 60% suppression of cardiac *Agtr2* expression in ZDF-F compared to ZL-F (Fig. 9a, p < 0.05), while there was no significant difference between the cardiac *Agtr2* expression of ZL-M and ZDF-M. Thus, the heart tissues of ZDF-F have a significant loss of cardio-protective *Agtr2* compared to ZL-F. Attenuation of cardiac *Agtr2* is a female- and diabetes-specific effect because healthy ZL-M displayed lower expression of cardiac *Agtr2* compared with ZL-F and levels in ZDF-M were not lowered with diabetes.

### MED13-miR-208a-axis differs between male and female diabetic animals

Cardiac Mediator Complex 13 (MED13), encoded by the *Med13* gene regulates systemic energy homeostasis, confers resistance to weight gain, and improves systemic insulin sensitivity and glucose tolerance^38,39^. As shown in Fig. 9b, cardiac expression of *Med13* was reduced by 50% (p < 0.05) in ZL-M compared to ZL-F. To our knowledge, this is the first report that shows sex bias in the expression of *Med13*. However, cardiac *Med13* expression was suppressed in both ZDF-F and ZDF-M rats compared to that of their lean counterparts (Fig. 9b, p < 0.05).

MED13 is a target of miR-208a, a microRNA that promotes cardiac hypertrophy and heart failure^38–41^. Since ZDF-F exhibited cardiac hypertrophy, we examined whether there was a sex bias in the expression of cardiac miR-208a. Interestingly, cardiac expression of miR-208a was higher in ZL-F compared to ZL-M and was the highest in ZDF-F (Fig. 9c, p < 0.05). Since increased expression of miR-208a is associated with cardiac hypertrophy, the female-specific increase in cardiac miR-208a expression could have contributed to the increased susceptibility to cardiac hypertrophy in ZDF-F.

### Cardiac miR-29 family miRNA expression patterns are different in male and female diabetic rats

Increases in the circulating levels of miR-29 family miRNAs in children with T1DM and adults with T2DM^42,43^ emphasize the clinical relevance of this biomarker in DM. We previously showed that increased miR-29 family miRNAs correlate with DM-induced cardiomyocyte disorganization^44^. Given the critical role of miR-29 in both DM and cardiac structure, we compared the cardiac expression of miR-29a, b and c between our groups. Cardiac expression of all members of the miR-29 family increased in both ZDF-F and ZDF-M compared to ZL-F and ZL-M (Fig. 9d–f, p < 0.05). Diabetes-induced increases in the levels of cardiac miR-29a and 29c were dependent upon the sex of the animal (Fig. 9d and f). Additionally, miR-29b expression was highest in ZDF-F (Fig. 9e). Thus, increases in the expression levels of the individual DM-associated miR-29 isoforms are also largely dependent on sex.

## Discussion

This study highlights the unique sex specific differences related to the progression of cardiac disease in the context of diabetes. We report that ZDF-F exhibited concentric cardiac hypertrophy and remodeling at 3-months (Fig. 3), and this cardiac hypertrophy persisted at 5-months (Fig. 8a). Importantly, we show that hypertrophic myocardial remodeling at both the gross and cellular levels occurred in ZDF-F and associated with an increase in structural damage (Fig. 8). There was a 66% increase in cardiomyocyte cross-sectional area in ZDF-F (Fig. 8b and d) In contrast, ZDF-M did not exhibit cardiomyocyte hypertrophy or increase in heart weight compared to ZL-M and this data is consistent with previous reports^13^. Both ZDF-F and ZDF-M suffered from loss of capillary density compared to their lean counterparts (Fig. 8c). However, only ZDF-F exhibited cardiac hypertrophy with reduced capillary density.

DM is a major contributing factor to heart failure. Conventional echocardiography cannot always uncover subclinical deformation of the heart. Early detection is key to preventing adverse cardiac events, and the sooner it is discovered, the better outcomes are for patients. However, two-dimensional speckle tracking echocardiography (STE) has emerged as a powerful tool to detect subclinical changes in asymptomatic patients suffering from heart disease^45–47^. Since diabetic females are at higher risk for heart disease, it is critical to identify factors that contribute to early changes in cardiac functions. We observed significant impairment of myocardial radial strain and strain rate in 3-month old ZDF-F compared to ZL-F (Fig. 3). Therefore, ZDF-F exhibits early development of impaired systolic mechanics that was not detected using conventional M-mode ultrasound. These observations are consistent with the hypothesis that there are early detectable changes in systolic function in the natural course of T2DM by STE study that are not detectable by traditional clinical indices of cardiac function such as ejection fraction^44,46,47^. A Previous study has shown a relationship between radial strain in the inner half layer of the LV wall and LV concentric hypertrophy^48^. Moreover, impaired radial strain is associated with myocardial histopathology in a rat model of doxorubicin-induced cardiomyopathy^49^ and detection of myocardial strain is a useful diagnostic approach to evaluate subtle cardiac changes in response to drugs.

An important finding from this study is that the progression of T2DM associated CVD in young hyperglycemic males involves cardiac fibrosis and this is not seen in young hyperglycemic females. In this context, we found a deficiency in several anti-fibrotic and anti-inflammatory cytokines in ZDF-M hearts compared to both ZDF-F- and ZL-M hearts, suggesting that T2DM-induced changes in intracardiac cytokines predisposed the male myocardium to fibrosis via the canonical immune response. The anti-fibrotic cytokines GM-CSF^50–52^ IL-10^53,54^ and IFN^55,56^ are suppressed in ZDF-M hearts. GM-CSF deficiency in the lungs increases fibrosis, while it also primes inflammatory dendritic cell formation, which is essential for initiation of a primary immune response^50–52^. Intracardiac IL-10 is nearly 2.0-fold less abundant in ZDF-M compared to ZDF-F or ZL-M. Moreover, IL-10 and IL-2^57^ are cardioprotective cytokines, produced primarily by monocytes and both are downregulated in ZDF-M hearts. Thus, T2DM progression causes a collective down regulation of anti-inflammatory and anti-fibrotic cytokines in male hearts, but not in female hearts. Finally, a reduction in pro-inflammatory cytokines such as MCP-1, IL-1β, IL-2, IFNγ, prolactin-R and IL-4 in ZDF-M indicates that T2DM promotes a pan-suppression immune response in male heart.

Estrogen enhances expression of IL-10 within the blood^58^, and IL-2 and IFN-γ mRNA in immune cells^59^. Promoters of IL-10 and IFN-γ contain estrogen response elements (ERE)^60–62^. Cardiac expression of these estrogen-regulated cytokines were similar in ZL-F and ZL-M. However, T2DM caused a suppression of IL-10, IL-2 and IFN-γ in male heart (ZL-M versus ZDF-M); but not in the pre-menopausal female heart (ZL-F versus ZDF-F). These data suggest that availability of estrogen in premenopausal ZL-F and ZDF-F could maintain expression of anti-fibrotic cardiac cytokines and that males do not have this protection. Conversely, expression of neuropilin, a cytokine that attenuates cardiomyopathy^63^, is higher in both ZL-M and ZDF-M compared to ZL-F and ZDF-F (Fig. 4). This sex bias in the expression of neuropilin may contribute to increased female-specific CVD risk.

Our data indicate that there is an uncoupling of hypertrophy and fibrosis in the heart that is dependent on the characteristics of DM and biological sex. We and others reported that in hyperinsulinemic and mildly hyperglycemic male Zucker Obese (ZO) rats, cardiac hypertrophy and fibrosis co-exist^21,64^. However, in ZDF-M with prolonged hyperglycemia and a short term hyperinsulinemia (due to pancreatic beta cell burn-out), neither cardiac hypertrophy nor mTOR activation are seen due to muscle wasting in response to severe DM, yet, they exhibit fibrosis. Conversely, hyperinsulinemic, hyperglycemic ZDF-F exhibit cardiac hypertrophy, but not fibrosis. These observations highlight the subtle differences in pathologic cardiac remodeling of males and females at different T2DM-stages that modulate the differences in their severity of CVD.

Activating AT2R signaling by agonists and increasing *Agtr2* gene copy number by genetic manipulation in murine models improves cardiac repair and enhances cardiac function^35–37^. The X-linked *Agtr2* that codes for AT2R is a 30-year old mechanistic target for treatment of CVD^32–37^. AT2R activation improves survival of mouse HL-1 cardiomyocytes and human coronary artery vascular smooth muscle cells under conditions of serum starvation^65^ and reduces infarct size after myocardial infarction in murine models^35,66^. Increased *Agtr2* expression in the female vasculature is implicated in the increased female-specific protection from hypertension, vascular injury, and renal function^32–34^. The data presented here show for the first time that T2DM suppresses cardiac *Agtr2* expression in a sex-biased manner affecting only female rats. (Fig. 9a).

Cardiac MED13 regulates systemic energy homeostasis, glucose tolerance, and resistance to weight gain^38,39^. Thus, MED13 is an important link between cardiac physiology, insulin resistance and obesity. Here we report a sex bias in cardiac expression of *Med13*. We propose that increased cardiac *Med13* mRNA expression in ZL-F may equip ZL-F to resist weight gain, and maintain insulin sensitivity and glucose tolerance better compared to ZL-M. Notably, ZDF-F do not develop hyperglycemia on normal rat chow whereas ZDF-M develop severe hyperglycemia on this diet. Therefore, we fed ZDF-F, with a high fat diet (diet#12468) to induce hyperglycemia. A previous study showed that that ZDF-M fed with diet#12468 had similar levels of diastolic dysfunction, and cardiovascular abnormalities, as observed in ZDF-M fed normal chow^22^. Therefore, diet #12468 is not a confounding factor when assessing cardiovascular disease outcomes.

Many MicroRNAs function as mechanistic biomarkers for CVD and contribute to cardiac damage. MicroRNA miR-208a promotes cardiac hypertrophy and heart failure^40,41^. Elevated levels of cardiac miR-208a expression in ZL-F and ZDF-F compared to ZL-M and ZDF-M could render female rats more susceptible to cardiac hypertrophy. Elevated miR-208a reduces *MED13* expression, thus contributing to obesity^38,39^. However, *Med13* expression was the highest in ZL-F, indicating that cardiac *Med13* mRNA expression in healthy ZL-F could be regulated by mechanisms independent of miR-208a. Importantly, reduction in cardiac *Med13* in response to T2DM in both sexes was similar (Fig. 9b), indicating its role in promoting cardiac insulin resistance in diabetes. Notably, though ZL-F has higher cardiac expression of pro-hypertrophic miR-208a, they also have higher expression of cardio-reparative *Agtr2* that may counteract miR-208a’s cardio-detrimental effect and keep the heart healthy. In ZDF-M, cardiac miR-208a expression was higher than that in ZL-M, yet lower than ZL-F and ZDF-F. Moreover, in ZDF-M cardiac *Agtr2* expression was similar to ZL-M. Conversely, in ZDF-F, cardiac miR-208a expression was increased while *Agtr2* expression was reduced. Thus, T2DM caused an imbalance in cardiac miR-208a-*Agtr2* expression pattern that would exacerbate cardiac damage.

Members of the microRNA miR-29 family (miR-29a, b, and c) serve as mechanistic biomarkers for diabetes (T1DM and T2DM) and cardiovascular damage. miR-29a is a blood biomarker in humans for hypertrophic cardiomyopathy and fibrosis^67^. miR-29b is implicated in the development of early aortic aneurysm^68^, whereas miR-29c is considered as a signature molecule of hyperglycemia^69^. We showed that elevated miR-29 family miRNAs correlate with increased cardiomyocyte disarray in 15-week old ZDF-M^44^. Here we show for the first time that while all miR-29 family miRNAs increased in response to diabetes in both sexes, there was a sex difference in their expression patterns. Differential expression of miR-29b was highest between ZDF-F and ZL-F, while differential expression of cardiac miR-29a and c were prominent between ZDF-M and ZL-M.

**Figure 10:**
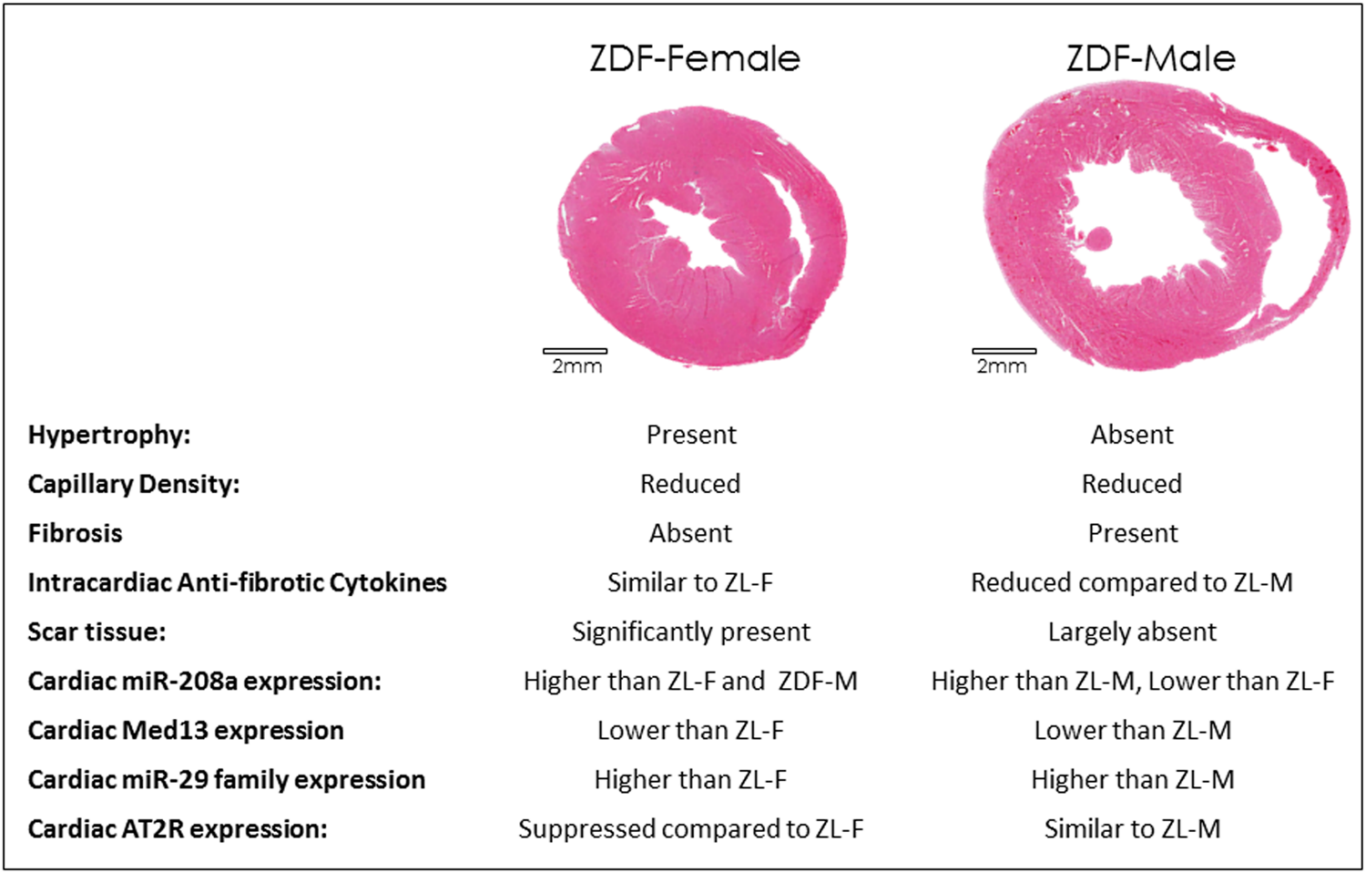
Differences between cardiac pathology of male and female diabetic hearts and mechanisms that may contribute to the higher susceptibility of ZDF-F heart to myocardial damage. Haematoxylin and eosin (H&E) stained cardiac sections of ZDF-F and ZDF-M show differences between the architecture of ZDF-M heart that does not have hypertrophy and the hypertrophied ZDF-F heart. Female specific increased expression of cardiac miR-208a (as shown in Fig. 9) could render ZDF-F rats more susceptible to hypertrophy. However, intracardiac cytokine profile analysis as shown in Figs 4–7 suggested that ZDF-M heart has fibrosis and suppression of anti-fibrotic cytokines, but ZDF-F heart did not exhibit this pathology. As shown in Fig. 8h, both ZDF-F and ZDF-M hearts have ultrastructural damage, however, only ZDF-F heart exhibited regions of scar tissue indicating increased myocardial damage. DM-associated dysregulation of miR-208a-MED13 signaling and increase in miR-29 family miRNAs occur in both male and female ZDF rats, however, only ZDF-F rats exhibited myocardial damage indicating that cardiac repair is impaired in ZDF-F. It is conceivable that the higher expression of cardio-reparative *Agtr2* in ZL-F compared to ZL-M (Fig. 9a) could have provided increased protection despite the higher expression of pro-hypertrophic miR-208a in ZL-F heart compared to ZL-M heart. However, reduction in cardiac *Agtr2* expression in ZDF-F in the presence of the highest expression of pro-hypertrophic miR-208a may have contributed to the increased myocardial structural damage observed in ZDF-F rat. Therefore, miR-208a and AT2R can be potential therapeutic targets for CVD in diabetic females.

Collectively our data show that T2DM-associated CVD progression in ZDF-F and ZDF-M is structurally and mechanistically different. ZDF-M exhibits cardiac fibrosis and significant suppression of intra-cardiac anti-fibrotic and anti-inflammatory cytokines. Conversely, ZDF-F hearts are protected from loss of anti-fibrotic cytokines and do not develop fibrosis. However, they exhibit cardiac and cardiomyocyte hypertrophy, as indicated via increased phosphorylation of mTOR. Though reduction in capillary density was a common phenomenon in both ZDF-F and ZDF-M, this effect is likely more deleterious for the hypertrophied myocardium of ZDF-F. It is conceivable that cardiac hypertrophy, capillary rarefaction, and a female-specific loss of cardio-reparative *Agtr2* in the setting of a very high expression of pro-hypertrophic miR-208a could have contributed to increased myocardial structural damage in the form of cardiomyocyte loss and scarring as shown in Fig. 8f. Factors identified in this study that may contribute to differences in the progression of T2DM-associated CVD in male and female rats are listed in Fig. 10. Our observations underscore the need for clinically expanding existing cardiac assessments in diabetic female patients to detect myocardial deformation, cardiac hypertrophy and capillary density via non-invasive imaging, as well as suggest miR-208a and AT2R as potential therapeutic targets for cardiac disease in females.

## Methods

### Animals, EchoMRI, and fasting plasma profile

All animal procedures used in this study were approved prior to the beginning of these studies by the Harry S. Truman Veterans Memorial Hospital (HSTVMH) Subcommittee for Animal Safety and University of Missouri IACUC. All animals were cared for in accordance with the Guidelines for the Care and Use of Laboratory Animals (National Institutes of Health publication 85–23). To generate a hyperglycemic ZDF-F rat, we fed female ZDF rats with diabetogenic diet (Diet#12468) starting at 6-weeks of age. Body composition in male and female ZL (ZL-F) and ZDF-F rats was determined using EchoMRI 4in1/1100 as described previously^44^. Levels of fasting plasma glucose, and serum insulin and triglycerides were measured as described previously^44^. The animals used in this study were young, breeding age (6 to 19 weeks of age) and any functional differences in female animals are not due to estrogen depletion from menopause or ovariectomy. While ZDF-M develops hyperglycemia on normal rat chow (Diet #5008), ZDF-F does not develop hyperglycemia on this diet, despite being hyperphagic and obese. However, this female-specific protection from hyperglycemia is lost when they are fed with a high fat diabetogenic diet (Diet #12468 that contains 48% fat)^70,71^.

### Echocardiography

Transthoracic echocardiography of a cohort of animals at the age of 3- and 5-months was performed under inhaled isofluorane anesthesia (0.5–1.0% maintenance) with a 12-MHz pediatric transducer using a GE Vivid I Ultrasound system to assess *in vivo* cardiac morphology, diastolic and systolic function as previously described^72^. Additionally, echocardiography was also performed on another cohort of 3-month old ZL-F and ZDF-F rats utilizing a Vevo2100 dedicated rodent ultrasound imaging system (Visualsonics, Toronto) with an MS250 high frequency echo probe at the Small Animal Ultrasound Imaging Center at the Harry S Truman VA Research Center. Speckle-tracking based strain analysis of B-Mode ultrasound images was performed in the parasternal long- and short-axis views (PLAX and SAX, respectively). Images were acquired at the highest frame rates possible (200–330 frames per second). Quantitation of strain and strain rate were performed in the longitudinal, radial, and circumferential axes. PLAX views were used for evaluation of longitudinal strain and strain rate, while SAX views, acquired at the mid-papillary level, were used for evaluation of circumferential and radial strain analyses. Strain analyses were conducted offline utilizing the manufacturer supplied speckle-tracking algorithm (VevoStrain®, VisualSonics). Briefly, at least 3 of the highest quality B-mode loops were chosen, i.e., those with little gel artifact or obstruction from ribs, as well as those that display the endocardial and epicardial borders throughout the cine loop. Initially, the endocardial and epicardial borders were traced with the cine loop stopped at end diastole. Cine-loops were replayed to confirm good border tracking over all cardiac cycles and tracking adjustments were made as needed. The final tracked images were then evaluated for strain measurements. Strain measures were averaged over the cardiac cycles yielding curvilinear strain and strain rate data. Global strain values, peak strain and strain rate measurements of ZDF rats were compared to those of control ZL rats.

### Histopathology and Immunohistochemistry

Heart tissues were fixed in 10% neutral buffered formalin (NBF) and embedded into paraffin blocks. Five micrometer heart sections were dewaxed in CitriSolv (Fisher Scientific), rehydrated in an ethanol series and HEPES wash buffer, followed by a heat-mediated antigen retrieval step in sodium citrate buffer. To block non-specific binding sites, sections were incubated with blocking buffer (10% donkey serum, 1% BSA) for 2 h at room temperature, followed by incubation with *Helix pomatia* agglutinin (HPA) conjugated to Alexa Fluor 647 (Life Technologies; 1:400, 2.5 µg/mL) and *Griffonia simplicifolia* isolectin B4 (IB4) conjugated to Alexa Fluor 594 (Life Technologies; 1:200, 5 µg/mL) for 4 h at room temperature. Additional sections were prepared, rehydrated, and blocked as described above and used for COL1A staining (COL1A antibody – Abcam #34710, 1:250; secondary antibody, Thermo Fisher Alexa Fluor 647 Donkey anti-Rabbit IgG, 1:500). Sections were thoroughly washed and slides were mounted using Fluoroshield with DAPI (Sigma). Imaging was performed using a Leica DMI4000B inverted confocal microscope at 40X and 63X. Cross-sectional area (µm^2^) of cardiomyocytes were measured using ImageJ (NIH, Bethesda, MD). To assess capillary rarefaction, the ratio of capillaries to cardiomyocytes was calculated. Heart sections were also stained with haematoxylin and eosin (H&E) or Masson’s trichrome stain (MTS) as described previously^44^.

### Interstitial fibrosis measurements

Interstitial fibrosis was measured using Aperio ImageScope (Leica Biosystems, IL USA). Briefly, heart tissues were fixed in 10% neutral buffered formalin (Sigma, St. Louis MO) followed by paraffin embedding. Next, samples were sliced into 4 μm thick slices, and were stained with Masson’s Trichrome Stain (MTS) at Research Animal Diagnostic Laboratory (Idexx RADIL, Columbia MO). After scanning slides, Aperio ImageScope was used to quantify 10 interstitial images (20X) of the most fibrotic regions per sample. The built-in algorithm Positive Pixel Count (V9) was used with the following parameters to determine percent fibrosis (Hue value: 0.6875, Hue width: 0.4, Color saturation threshold: 0.0). Lastly, positivity (positive/total pixels) were averaged over all regions from a single group to determine interstitial fibrosis.

### Western blotting

To determine the changes in the phosphorylation status of serine-^2448^ of mTOR, cardiac tissue lysates were subjected to Western blotting as described previously^39,44^. mTOR and phospho-mTOR (Ser^2448^) antibody were purchased from Cell Signaling Technology, highlighted. Tris-buffered saline-Tween 20 (TBST) containing 5% bovine serum albumin (BSA) was used for blocking the Western blots (PVDF) for one hour. Primary antibodies were diluted 1:1000 in 5% BSA in TBST. Blots were incubated for overnight at 4 °C in primary antibodies, washed with TBST, and were incubated in the horseradish peroxidase-conjugated secondary antibody (1:25,000 dilution in 5% BSA in TBST). After TBST washes, chemiluminescent substrate (Supersignal West Femto Maximum Sensitivity Substrate kit; Thermo Scientific) was added to visualize antibody binding using Bio-Rad ChemiDoc XRS image-analysis system. Quantitation of pSer^2448^-mTOR band density compared to total mTOR protein band density was performed and ratios were calculated. All protein band density quantifications were performed using Quantity One software (Bio-Rad Laboratories Inc. Berkeley, Ca). Data are reported as the normalized protein band density in arbitrary units.

### Intracardiac cytokine analysis using Quantibody^®^ Rat Cytokine Array 67 and IPA analysis

Previously frozen (−80 °C) heart samples were homogenized under liquid nitrogen. Briefly, upon bead-homogenation with ice cold lysis buffer containing appropriate phosphatase and protease inhibitors, samples were centrifuged, and resultant protein concentrations were determined using the bicinchoninic acid (BCA) assay (Thermo Fisher Scientific, Waltham MN, USA). Next, 300 μg of homogenate of each sample was sent to RayBiotech and used for Quatibody Rat Cytokine Array 67. The assay is a combination of 2 non-overlapping arrays that results in quantitation of 67 unique cytokines using antibody pairs. The data is normalized and positive and negative controls allow for standard deviation and statistics to be performed. Cytokines that passed a statistical analysis of p < 0.05 between the different groups compared were put into the bioinformatics software Ingenuity Pathway Analysis (Qiagen, CA USA) in order to understand pathways and diseases effected.

### RNA isolation and quantitative Real Time-PCR

Cardiac expression of AT2R (*Agtr2*), *Med13*, miR-208a, and miR-29 family miRNAs were determined using mRNA and miRNA isolated from frozen heart tissues as described previously^44^. Real-time PCR reactions were performed in triplicate using either TaqMan Fast Universal PCR Master Mix (2X) (Applied Biosystems) or SYBRSelect Master Mix (2X) (Life Technologies), and the 7500 Fast Real-Time PCR system (Applied Biosystems). TaqMan MicroRNA Assays (Life Technologies) primers for miR-208a, miR-29a, b, c, and U6 snRNA were used for miRNA targets, and *Med13* and 18 S rRNA for mRNA targets. Primer sequences of 18 S, and *Agtr2* that were used in this study were the following:

18 S forward: CTGAGAAACGGCTACCACATC, 18 S reverse: TTGGATGGTTTAGTGAGGCC;

*Agtr2*, forward: ATGAAGGACAACTTCAGTTTTGCTGCCACCAGC,

*Agtr2* reverse: TTAAGACACAAAGGTGTCCATTTCTCTAAGAG.

### Ultrastructural analysis with transmission electron microscopy

Details of tissue fixation, embedding, sectioning, and staining were performed as described previously^73^.

### Statistics

Statistical analysis was performed using GraphPad Prism 7 (GraphPad Software, Inc., La Jolla, CA). For multiple comparisons, one- or two-way ANOVA, or two-way repeated measures ANOVA, followed by uncorrected Fisher’s LSD, was performed as appropriate, with main effects of sex (S), diabetes (D), or a Sex*Diabetes interaction (INT) noted where relevant. Unpaired two-tailed *t*-test was performed for pairwise comparisons. A *p*-value < 0.05 was deemed significant.

### Data Availability Statement

All relevant data is presented in the manuscript and supplementary materials.

## Electronic supplementary material

Supplementary Material
