# Supplementary material for: Cardiovascular disease progression in female Zucker Diabetic Fatty rats occurs via unique mechanisms compared to males
Source: Sci Rep. 2017 Dec 19;7:17823. doi: 10.1038/s41598-017-18003-8 (PMC5736602; doi:10.1038/s41598-017-18003-8)
Supplement: Supplementary file 1 — Supplementary Material [file 41598_2017_18003_MOESM1_ESM.pdf]

Supplementary Information:

Cardiovascular disease progression in female Zucker Diabetic Fatty rats occurs via unique mechanisms compared to males

Kelly Lum-Naihe[#,1,5], Ryan Toedebusch[#,1,4,5], Abuzar Mahmood[1,4,5], Jamal Bajwa[1,5], Terry Carmack[5], Senthil A. Kumar[1,], Sivakumar Ardhanari[1,], Vincent G. DeMarco[1, 5],

Craig A. Emter[2,4] and Lakshmi Pulakat*[1,3,4,5]

[#]Co-first authors

Departments of Medicine[1], Biomedical Sciences[2], Nutrition and Exercise Physiology[3], Dalton Cardiovascular Research Center[4], University of Missouri, and Harry S Truman VA Hospital[5], Columbia, MO

***Corresponding Author:**

Dr. Lakshmi Pulakat
Department of Medicine and Department of Nutrition and Exercise Physiology
Dalton Cardiovascular Center
University of Missouri School of Medicine
Columbia, MO 65212
Email:pulakatl@health.missouri.edu

Supplemental Table S1. Comparison of systolic and diastolic function of 3- and 5-month old male and female ZL and ZDF rats.

	ZL-F		ZDF-F		ZL-M		ZDF-M		2x2 RM ANOVA
	3 mos.	5 mos.	3 mos.	5 mos.	3 mos.	5 mos.	3 mos.	5 mos.	
	SYSTOLIC FUNCTION								
**HR (beats/min)**	312 ± 10	301 ± 10	373 ± 11	349 ± 9	307 ± 9	347 ± 11	280 ± 10	266 ± 4	INT, ME-sex
**FS (%)**	57 ± 3	58 ± 1	53 ± 2	65 ± 4	45 ± 2	52 ± 2	50 ± 1	51 ± 1	INT, ME-sex
**LV SV (μL)**	378 ± 45	450 ± 30	314 ± 34	410 ± 23	425 ± 31	479 ± 25	481 ± 27	447 ± 55	ME-sex
**LVAWs (mm)**	2.8 ± 0.2	3.1 ± 0.2	3.1 ± 0.2	3.4 ± 0.1	3.0 ± 0.1	3.4 ± 0.1	3.1 ± 0.1	3.4 ± 0.1	
**LVPWs (mm)**	2.2 ± 0.1	2.7 ± 0.2	2.5 ± 0.1	3.4 ± 0.3	2.4 ± 0.1	2.7 ± 0.1	2.5 ± 0.1	3.0 ± 0.1	ME-diabetes
**LVIDs (mm)**	2.8 ± 0.4	2.5 ± 0.2	3.2 ± 0.3	2.1 ± 0.2	3.8 ± 0.2	3.2 ± 0.2	3.9 ± 0.1	3.5 ± 0.1	ME-sex
**S' (cm/sec; septum)**	3.4 ± 0.1	3.5 ± 0.1	3.7 ± 0.4	3.2 ± 0.1	3.5 ± 0.2	3.3 ± 0.2	3.4 ± 0.1	3.6 ± 0.2	
	DIASTOLIC FUNCTION								
**LVAWd (mm)**	1.6 ± 0.2	1.9 ± 0.2	1.9 ± 0.1	2.1 ± 0.2	1.8 ± 0.1	2.0 ± 0.1	1.7 ± 0.1	2.1 ± 0.1	
**LVPWd (mm)**	1.3 ± 0.1	1.5 ± 0.2	1.3 ± 0.1	2.0 ± 0.2	1.4 ± 0.1	1.9 ± 0.1	1.5 ± 0.1	2.0 ± 0.1	ME-sex, diabetes
**LVIDd (mm)**	6.3 ± 0.4	6.0 ± 0.3	6.7 ± 0.3	6.0 ± 0.3	6.9 ± 0.2	6.7 ± 0.1	7.7 ± 0.2	7.1 ± 0.1	ME-sex, diabetes (p=0.055)
**IVRT (msec)**	18 ± 1	18 ± 1	16 ± 1	20 ± 1	17 ± 1	17 ± 1	22 ± 1	27 ± 1	INT
**E (cm/sec)**	119 ± 9	125 ± 8	123 ± 6	115 ± 5	105 ± 4	119 ± 5	119 ± 3	123 ± 5	
**A (cm/sec)**	67 ± 3	73 ± 2	101 ± 8	92 ± 3	75 ± 4	91 ± 8	91 ± 7	100 ± 6	INT, ME-diabetes
**E/A ratio**	1.81 ± 0.14	1.71 ± 0.11	1.24 ± 0.05	1.25 ± 0.05	1.41 ± 0.06	1.32 ± 0.06	1.35 ± 0.09	1.20 ± 0.05	INT
**E' (cm/sec; septum)**	5.5 ± 0.3	5.9 ± 0.5	5.5 ± 0.2	6.1 ± 0.2	4.9 ± 0.3	6.3 ± 0.3	5.0 ± 0.3	5.4 ± 0.2	
**A' (cm/sec; septum)**	2.9 ± 0.1	4.1 ± 0.4	4.0 ± 0.3	3.7 ± 0.3	3.3 ± 0.3	4.5 ± 0.2	4.0 ± 0.2	4.3 ± 0.3	
**E'/A' ratio**	1.9 ± 0.1	1.5 ± 0.1	1.4 ± 0.1	1.7 ± 0.2	1.5 ± 0.1	1.4 ± 0.1	1.2 ± 0.1	1.3 ± 0.1	ME-sex, diabetes
**Relative wall thickness**	0.42 ± 0.07	0.50 ± 0.05	0.40 ± 0.02	0.67 ± 0.09	0.42 ± 0.03	0.55 ± 0.02	0.40 ± 0.01	0.57 ± 0.03	ME-diabetes

Values are means ± SEM. HR, heart rate; FS , fractional shortening; LV SV, left ventricular stroke volume; LVAWs, left ventricular systolic anterior wall thickness; LVPWs, left ventricular systolic posterior wall thickness; LVIDs, left ventricular internal systolic diameter; S', peak mitral annulus velocity during systole; LVAWd, left ventricular diastolic anterior wall thickness; LVPWd, left ventricular diastolic posterior wall thickness; LVIDd, left ventricular internal diastolic diameter; IVRT, isovolumic relaxation time; E, peak mitral flow velocity during early diastole; A, peak mitral flow velocity during atrial systole; E', early diastolic peak mitral annulus velocity; A', peak mitral annulus velocity during atrial systole. Stats: INT – Sex*DM interaction; ME – Main Effect of Sex or DM

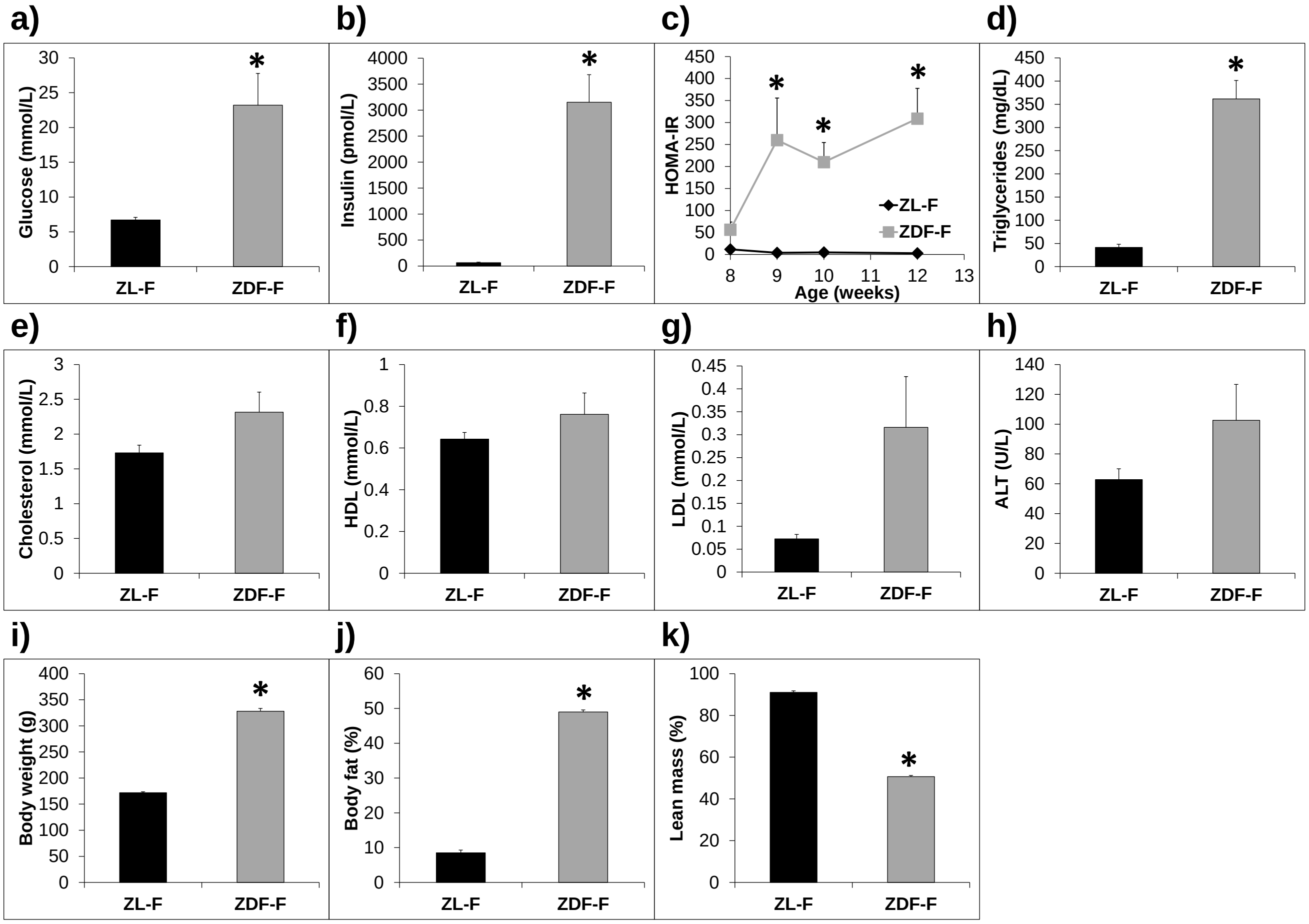

Supplemental Fig. S1. ***Characterization of the metabolic phenotype in 3-month old ZL-F and ZDF-F rats***.

Six-hour fasting blood collection at 3-months of age was performed to analyze (A) plasma glucose, (B) serum insulin, (D) triglycerides, (E) cholesterol, (F) high-density lipoprotein (HDL), (G) low-density lipoprotein (LDL) and (H) alanine aminotransferase (ALT). (C) HOMA-IR was calculated at the indicated ages using corresponding fasting plasma glucose and insulin values. (I) Body weight was measured at 3-months of age and (J and K) body composition was determined using the EchoMRI 4in1/1100, a QMR system that measures lean mass, fat mass, total water, and free water, in which rats were placed in an adjustable plastic cylinder to restrict movement. The cylinder (2.75 inches in diameter) has openings on either end to allow the animals to breathe freely. The cylinder was inserted into the EchoMRI for a reading that lasted for 85 seconds. Number of animals per group ranged from 8-11. Values are means ± SEM. n=5 for ZL-F and ZDF-F. *$p<0.05$ vs. ZL-F by unpaired *t*-test.

Supplemental Table S2. Echocardiography parameters of 3-month old ZL and ZDF female rats.

Parameter		ZL-F	ZDF-F	*p*-value
SAX, M-Mode				
Systolic function				
Heart Rate (bpm)		365 ± 9	364 ± 10	0.9472
Stroke Volume (µL)		192 ± 9	197 ± 6	0.6681
EF%		81.05 ± 1.46	84.15 ± 1.60	0.1253
FS%		51.21± 1.52	55.39 ± 1.88	0.0982
Diastolic function				
LVAWd (mm)		1.71 ± 0.08	1.94 ± 0.06	0.04
LVIDd (mm)		6.67 ± 0.15	6.72 ± 0.11	0.81
IVRT (ms)		16.99 ± 1.07 (10)	19.87 ± 1.30 (10)	0.10
E (mm/s)		1003.18 ± 46.41 (11)	875.42 ± 28.85	0.03
E' (mm/s)		67.72 ± 3.50 (11)	48.22 ± 2.33	<0.01
Myocardial strain				
Endocardial SAX				
	Radial strain	24.28 ± 2.66 (11)	15.91 ± 2.15 (11)	0.02
	Radial strain rate, 1/s	5.15 ± 0.35 (11)	3.75 ± 0.35 (11)	0.01
	Circumferential strain	-22.71 ± 1.71	-25.08 ± 2.56	0.45
	Circumferential strain rate, 1/s	-5.00 ± 0.40	-7.16 ± 0.78	0.06
Endocardial PLAX				
	Radial strain	24.15 ± 1.58 (10)	9.19 ± 1.88 (8)	<0.01
	Radial strain rate, 1/s	4.84 ± 0.36 (11)	3.50 ± 0.32 (11)	0.01
	Longitudinal strain	-17.49 ± 2.09	-15.42 ± 2.02	0.48
	Longitudinal strain rate, 1/s	-5.39 ± 0.61	-4.51 ± 0.38	0.23
Epicardial SAX				
	Circumferential strain	-8.83 ± 0.74	-7.14 ± 0.68 (11)	0.11
	Circumferential strain rate, 1/s	-2.03 ± 0.17	-1.88 ± 0.13	0.50
Epicardial PLAX				
	Longitudinal strain	-9.58 ± 0.78 (9)	-7.04 ± 0.62 (9)	0.02
	Longitudinal strain rate, 1/s	-2.07 ± 0.27	-2.07 ± 0.17	0.99
n=12 unless otherwise indicated in parentheses				

EF, Ejection Fraction; FS, Fractional Shortening, LVAWd, left ventricular diastolic anterior wall thickness; LVIDd, left ventricular internal diastolic diameter; IVRT, isovolumic relaxation time; E, peak mitral flow velocity in early diastole; E', early diastolic peak mitral annulus velocity; SAX: Short Axis view; PLAX: Parasternal long-axis view. *p*-values are listed and were determined by unpaired *t*-test.

Supplemental Table S3 Chemokine/cytokine fold change values as determined by Ray Biotech Quantibody-Rat Cytokine Array Q67. ZL-F = Zucker lean-female, ZL-M = Zucker lean-male, ZDF-F = Zucker diabetic fatty-female, ZDF-M = Zucker diabetic fatty-male. **Bolded** values indicate significance between respective groups compared ($p < 0.05$) via pairwise Student T-test.

Chemokine/cytokine	Fold-change ZL-F/ZL-M	Fold-change ZDF-F/ZDF-M	Fold-change ZDF-F/ZL-F	Fold-change ZDF-M/ZL-M	Classification
4-1BB	-1.67	2.55	-1.03	-4.39	Receptor
Activin A	---	5.07	---	-21.26	Receptor
Adiponectin	-1.44	2.02	-1.06	-3.07	Adipokine
b-NGF	2.15	1.52	-1.38	1.02	Growth Factor
B7-1	1.23	1.48	-1.51	-1.82	Receptor
B7-2	**5.96***	**4.22***	1.21	1.70	Receptor
CD48	-1.29	5.47	1.66	-4.26	Receptor
CINC-1	2.16	2.35	1.35	1.23	CXC Chemokine
CINC-2	**3.31***	**2.55***	1.29	1.67	CXC Chemokine
CINC-3	**4.97***	3.97	-1.80	-1.43	CXC Chemokine
CNTF	-1.47	1.22	1.11	-1.61	Cytokine
CTACK	-1.16	2.64	1.48	-2.08	CC Cytokine
Decorin	1.15	1.03	**-1.29***	-1.16	Ligand
Eotaxin	-1.49	1.53	1.24	-1.84	CC chemokine
EphA5	5.48	1.01	-2.99	1.81	Receptor
Erythropoietin	2.72	1.78	-1.34	1.14	Cytokine
FGF-BP	1.41	1.25	-1.61	-1.43	Growth Factor
Flt-3L	-2.02	1.18	-1.50	-3.55	Ligand
Fractalkine	1.71	1.05	-1.24	1.31	Chemokine
Galectin-1	1.52	1.00	-1.37	1.11	Receptor
Galectin-3	**2.04***	1.48	1.17	1.61	Receptor
Gas 1	**-2.85***	-1.03	-1.09	**-3.01***	Tumor Suppressor
GFR alpha-1	-1.07	-1.20	-1.18	-1.05	Receptor
GM-CSF	1.00	1.46	-1.13	**-1.64***	Cytokine
gp130	1.06	4.28	3.42	-1.17	Receptor
HGF	1.14	2.77	1.10	-2.21	Growth Factor
ICAM-1	-1.25	1.16	1.03	-1.41	Receptor
IFNg	1.09	1.63	-1.08	**-1.61***	Cytokine
IL-1 R6	4.46	-1.41	-2.00	3.14	Receptor
IL-1 ra	1.49	1.69	1.34	1.18	Cytokine
IL-10	1.07	**2.09***	1.11	**-1.75***	Cytokine
IL-13	4.91	2.16	-1.08	2.11	Cytokine
IL-17F	-1.06	-1.40	-3.95	-3.00	Cytokine
IL-1a	5.31	2.50	-1.19	1.78	Cytokine
IL-1b	1.13	1.61	-1.27	**-1.82***	Cytokine
IL-2	1.32	**2.28***	1.07	**-1.61***	Cytokine
IL-2 Ra	1.32	1.65	-1.50	-1.87	Receptor
IL-22	-172.65	---	---	---	Cytokine
IL-3	5.05	-1.83	-4.72	1.96	Cytokine
IL-4	1.11	1.57	-1.28	**-1.82***	Cytokine
IL-6	1.70	-1.15	**-2.50***	-1.28	Cytokine
IL-7	0.97	-1.09	-1.35	-1.29	Cytokine
JAM-A	1.23	-1.01	-1.19	1.05	Adhesion Molecule
L-Selectin	**3.98***	2.78	-1.07	1.34	Adhesion Molecule
LIX	**3.03***	1.48	-1.45	1.42	CXC Chemokine
MCP-1	1.05	1.52	-1.15	**-1.67***	CC Chemokine
MIP-1a	---	---	---	---	CC Chemokine
Neuropilin-1	**-1.27***	**-1.26***	**-1.68***	**-1.69***	Receptor
Neuropilin-2	-1.26	-1.05	-1.68	-2.01	Receptor

Nope	1.22	-1.32	-1.54	1.04	Adhesion Molecule
Notch-1	1.11	1.03	-1.50	-1.40	Receptor
Notch-2	-1.43	-1.48	-2.33	-2.26	Receptor
P-Cadherin	-1.26	4.93	6.77	1.09	Adhesion Molecule
PDGF-AA	3.01	1.07	-2.56	1.09	Growth Factor
Prolactin	1.74	1.93	-1.46	-1.62	Growth Factor
Prolactin R	1.52	**2.48***	1.08	-1.51	Receptor
RAGE	-1.82	8.34	1.12	-13.59	Receptor
RANTES	1.11	1.95	-1.93	-3.39	CC Chemokine
SCF	1.13	1.91	-1.54	-2.60	Growth Factor
TCK-1	1.16	-1.02	-1.24	-1.05	CXC Chemokine
TIM-1	1.24	1.31	-1.03	-1.08	Receptor
TIMP-1	-1.35	**2.79***	1.61	**-2.35***	MMP Inhibitor
TIMP-2	---	---	---	---	MMP Inhibitor
TNFa	1.11	1.70	-1.24	-1.91	Growth Factor
TREM-1	2.03	1.99	-1.10	-1.08	Receptor
TWEAK R	-1.09	1.06	-1.15	-1.33	Receptor
VEGF	**4.68***	1.63	-2.23	1.28	Growth Factor
